# Molecular Computational Anatomy: Unifying the Particle to Tissue Continuum via Measure Representations of the Brain

**DOI:** 10.34133/2022/9868673

**Published:** 2022-11-07

**Authors:** Michael Miller, Daniel Tward, Alain Trouvé

**Affiliations:** 1 Department of Biomedical Engineering & Kavli Neuroscience Discovery Institute & Center for Imaging Science, Johns Hopkins University, Baltimore, USA; 2 Departments of Computational Medicine & Neurology, University of California Los Angeles, Los Angeles, USA; 3 Centre Giovanni Borelli (UMR 9010), Ecole Normale Supérieure Paris-Saclay, Université Paris-Saclay, Gif-sur-Yvette, France

## Abstract

**Objective.:**

The objective of this research is to unify the molecular representations of spatial transcriptomics and cellular scale histology with the tissue scales of computational anatomy for brain mapping.

**Impact Statement.:**

We present a unified representation theory for brain mapping based on geometric varifold measures of the microscale deterministic structure and function with the statistical ensembles of the spatially aggregated tissue scales.

**Introduction.:**

Mapping across coordinate systems in computational anatomy allows us to understand structural and functional properties of the brain at the millimeter scale. New measurement technologies in digital pathology and spatial transcriptomics allow us to measure the brain molecule by molecule and cell by cell based on protein and transcriptomic functional identity. We currently have no mathematical representations for integrating consistently the tissue limits with the molecular particle descriptions. The formalism derived here demonstrates the methodology for transitioning consistently from the molecular scale of quantized particles—using mathematical structures as first introduced by Dirac as the class of generalized functions—to the tissue scales with methods originally introduced by Euler for fluids.

**Methods.:**

We introduce two mathematical methods based on notions of generalized functions and statistical mechanics. We use geometric varifolds, a product measure on space and function, to represent functional states at the micro-scales—electrophysiology, molecular histology—integrated with a Boltzmann-like program to pass from deterministic particle descriptions to empirical probabilities on the functional states at the tissue scales.

**Results.:**

Our space-function varifold representation provides a recipe for traversing from molecular to tissue scales in terms of a cascade of linear space scaling composed with nonlinear functional feature mapping. Following the cascade implies every scale is a geometric measure so that a universal family of measure norms can be introduced which quantifies the geodesic connection between brains in the orbit independent of the probing technology, whether it be RNA identities, Tau or amyloid histology, spike trains, or dense MR imagery.

**Conclusions.:**

We demonstrate a unified brain mapping theory for molecular and tissue scales based on geometric measure representations. We call the consistent aggregation of tissue scales from particle and cellular scales, molecular computational anatomy.

## 1. Introduction

One of the striking aspects of the study of the brain in modern neurobiology is the fact that the distributions of discrete structures that make up physical tissue, from neural cells to synapses to genes and molecules, exist across nearly ten orders of magnitude in spatial scale. This paper focuses on the challenge of building multiscale representations that simultaneously connect the quantized nanoscales of modern molecular biology and digital pathology for characterizing neural circuit architecture in the brain with the classical continuum representations at the anatomical gross and mesoscales.

We have been highly motivated by the Cell Census Network project (BICCN [1]) which highlights the interplay between the nano- and micron scales of single-cell measures of RNA via spatial transcriptomics [2–4] coupled to the tissue scales of mouse atlases. The recent review on bridging scales from cells to physiology [5] motivates the mathematical framework presented herein. The recent emergence of spatial transcriptomics as 2020 Nature Method of the Year highlights the importance of such approaches for understanding the dense metric structure of the brain built up from dense imaging measurements at the cellular scales. Specifically, in our own work on digital pathology for the study of Alzheimer’s disease called the BIOCARD study [6], we are examining pathological Tau in the medial temporal lobe (MTL) at both the microhistological and macroscopic atlas scales, from 10 to 100*μ*m [7, 8], extended to the magnetic resonance millimeter scales for examining entire circuits in the MTL. In the mouse cell census project, we are examining single-cell spatial transcriptomics using modern RNA sequencing in dense tissue at the micron scale and its representations in the Allen atlas coordinates [9].

Most noteworthy for any representation is that at the finest microscales, nothing is smooth; the distributions of cells and molecules are more well described as random quantum counting processes in space [10]. In contrast, information associated to atlasing methods at the gross anatomical tissue and organ scales of computational anatomy extend smoothly [11–16]. Cross-sectionally and even cross-species, gross anatomical labelling is largely repeatable, implying information transfers and changes from one coordinate system to another smoothly. This is built into the representation theory of diffeomorphisms and soft matter tissue models for which advection and transport hold [17–23], principles upon which continuum mechanics and computational anatomy are based. Also of note is the fact that the brain organizes information on geometric objects, submanifolds of the brains such as the foliation of the cortex and associated coordinates of the cortical columns. Our representations must both represent the quantum to ensemble scales and encode the global macroscopic organizations of the brain.

The focus of this paper is to build a coherent representation theory across scales. For this, we view the micron to millimeter scales via the same representation theory called mathematical *geometric measures*, building the finest micron scales from discrete units termed varifold measures which represent space and the function of molecules, synapses, and cells. The measure representation from fine- to coarse-scale aggregates forming tissue. This measure representation allows us to understand subsets of tissues that contain discretely positioned and placed functional objects at the finest quantized scales and simultaneously pass smoothly with aggregation to the classical continuum scales at which stable functional and anatomical representations exist. Since the study of the function of the brain on its geometric submanifolds—the gyri, sulci, subnuclei, and laminae of the cortex—is so important, we extend our general framework to exploit varifold measures [24] arising in the modern discipline of geometric measure theory. Varifolds are defined as a cross-product measure on space with a measure on molecular function. Geometric measures are a class of generalized functions which have the basic measure property of additivity on disjoint unions of the experimental probe space and encode the complex physiological functions with the geometric properties of the submanifolds to which they are associated. To be able to compare the brains, we use diffeomorphisms as the comparator tool, with their action representing 3D varifold action which we formulate as “copy and paste” so that basic particle quantities that are conserved biologically are combined with greater multiplicity and not geometrically distorted as would be the case for measure transport.

The functional features are represented via generalized Dirac delta functions at the finest microstructure scales. The functional feature is abstracted into a function space rich enough to accommodate the molecular machinery as represented by RNA or Tau particles, as well as electrophysiology associated to spiking neurons, or at the tissue scales of medical imaging dense contrasts of magnetic resonance images (MRIs). We pass to the classical function continuum via introduction of a scale space that extends the descriptions of cortical microcircuits to the meso- and anatomical scales. This passage from the quantized features to the stochastic laws is in fact akin to the Boltzmann program transferring the view from the Newtonian particles to the stable distributions describing them. For this, we introduce a scale space of kernel density transformations which allows us to retrieve the empirical averages represented by the determinism of the stochastic law consistent with our views of the macroscopic tissue scales.

The representation provides a recipe for scale traversal in terms of a cascade of linear space scaling composed with nonlinear functional feature mapping. Following the cascade implies every scale is a varifold measure so that a universal family of varifold norms can be introduced which simultaneously measure the disparity between brains in the orbit independent of the probing technology, yielding one of the many types of data: RNA identities, Tau or amyloid histology, spike trains, or dense MR imagery.

Our multiscale brain measure model implies the existence of a sequence. We call this scale space of pairs, the measure representation of the brain, and the associated probing measurement technologies Brainspace. To formulate a consistent measurement and comparison technology on Brainspace, we construct a natural metric upon it allowing us to study its geometry and connectedness. The metric between brains is constructed via a Hamiltonian which defines the geodesic connections throughout scale space, providing for the first time a hierarchical representation that unifies microscopic to millimeter representation in the brain and makes Brainspace into a metric space. Examples of representation and comparison are given for Alzheimer’s histology integrated to magnetic resonance imaging scales and spatial transcriptomics. We call the consistent formalism presented here for aggregation of tissue scales from particle and cellular scales Molecular Computational Anatomy.

## 2. Results

### 2.1. Measure Model of Brain Structures.

**Figure 1:**
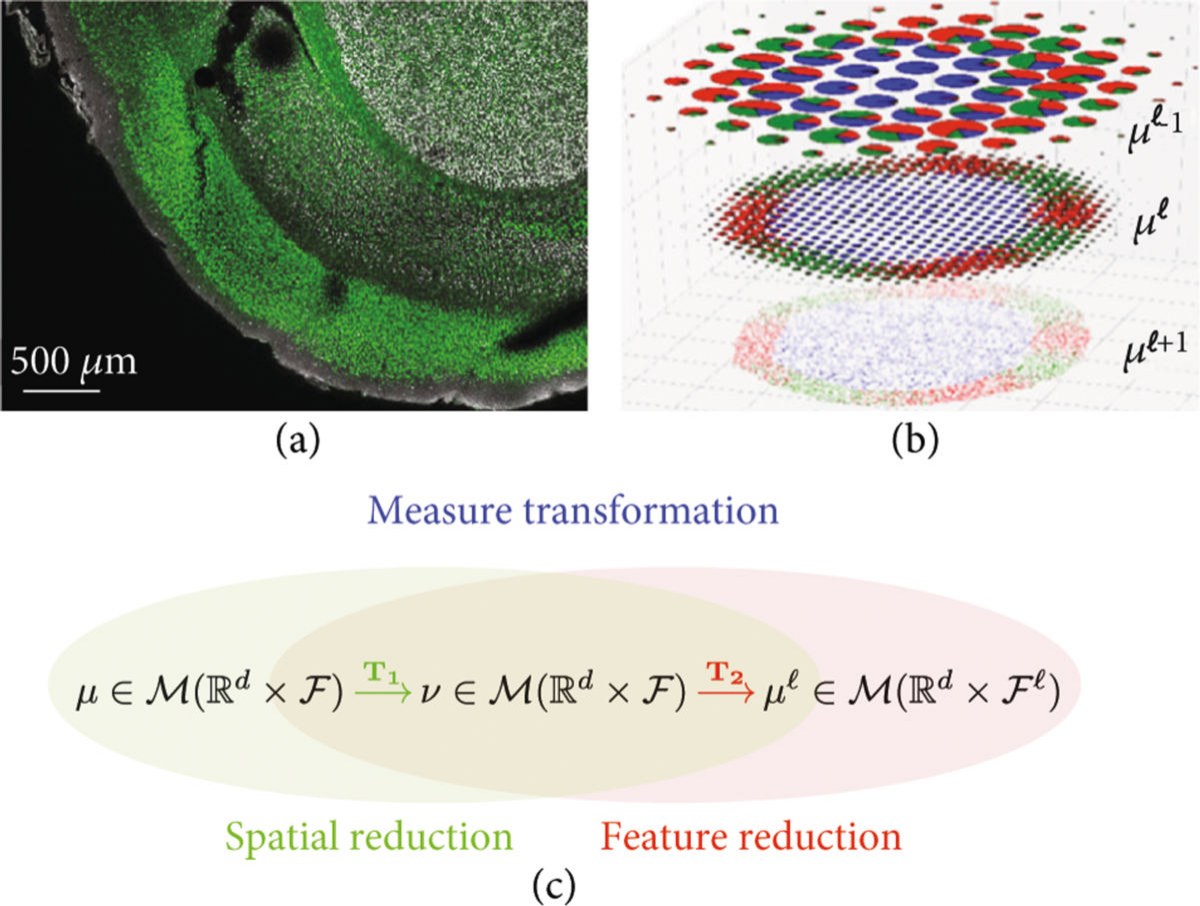
(a) Tissue from a NexCre+/−; Bm2fi/+ adult mouse mimicking a wild-type mouse with CUXl labelling of layers ii/iii and iv and CTIP2 in layers v and vi in green. It shows sections at 2:5^2^ × 50 *μ*m^3^ 6 tile images, 1433 × 1973 pixels; taken from Uli Mueller. (b) Shows the abstraction of a coarse-to-fine hierarchy $\mu^{\ell -1},\mu^{\ell },\mu^{\ell +1}$ with fine molecular scales shown at the bottom with colors depicting ℱ function ascending scales. (c) Space and function transformation shown as a composition $\mu \overset{T_{1}}{\to }v\overset{T_{2}}{\to }\mu^{\ell }$.

To build a coherent theory, we view the micron to anatomical scales via the same representation theory building upon discrete units termed particles or atoms. As they aggregate, they form tissues. This is depicted in Figure 1 in which (a) shows mouse imaging of CUX1 labelling of the inner layers of mouse cortex (white) and CTP2 imaging of the outer layers (green) at 2.5 micron in plane resolution. Notice the discrete nature of the cells clearly resolved which form the layers of tissue which are the global macro scale features of layers 2, 3, and 4 which stain more prolifically in white and the outer layers 5 and 6 which stain more prolifically in green.

Our representation exists simultaneously at both the microscopic and tissue millimeter scales. A key aspect of anatomy is that at the microscale, information is encoded as a massive collection of pairs $\left(x_{i},f_{i}\right)$ where $x_{i}\in {\mathbb{R}}^{d}(d=2,3)$ describes the position of a “particle” and $f_{i}$ is a functional state in a given set ℱ attached to it. In our applications, ℱ are proteins representing RNA signatures or Tau tangles and for single-cell neurophysiology represent the dynamics of neural spiking. At the microscale, basically, everything is deterministic, with every particle attached to its own functional state among possible functional states in ℱ. But zooming out, the tissue level, say millimeter scale, appears through the statistical distribution of its constituents with two key quantities, the local density of particles and the conditional probability distribution of the functional features $\mu_{x}(df)$ at any location x. At position x, we no longer have a deterministic functional state but a distribution $\mu_{x}$ on functional states, which we represent analogous to the Boltzman probability.

The integration of both descriptions into a common mathematical framework can be done quite naturally in the setting of mathematical measures which are mathematical constructs that are able to represent both the discrete and continuous worlds as well as the natural levels of approximation between both.

At the finest scale we associate to particles the elementary “Dirac” $\delta_{x_{i}}⊗\delta_{f_{i}}$ which applies to infinitesimal volumes in space dx and function df so that it evaluates as $\delta_{x_{i}}(dx)\delta_{f_{i}}(df)$, which is equal to 1 if $x_{i}\in dx$ and $f_{i}\in df$, and 0 otherwise. Indeed, the set $ℳ\left({\mathbb{R}}^{d}\times ℱ\right)$ of finite positive measures on ${\mathbb{R}}^{d}\times ℱ$ contains discrete measures written as

(1) $$\mu =\sum_{i\in I}w_{i}\delta_{x_{i}}⊗\delta_{f_{i}},$$

where $w_{i}$ is a positive weight, that can encode the collection ${\left(x_{i},f_{i}\right)}_{i\in I}$ at microscale.

As in Boltzmann modeling, we describe the features statistically at a fixed spatial scale transferring our attention to their stochastic laws modeled as conditional probabilities in ${ℳ}_{P}(ℱ)$ with integral 1. For this, we factor the measures into the marginal space measure $\rho^{\mu }$ on ${\mathbb{R}}^{d}$ with $\rho^{\mu }(dx)=\int_{ℱ}\mu (dx,df)$, and the field of probability distributions on ℱ conditioned on x. For the convention dx and df taken as events gives the factorization

(2) $$\mu (dx,df)=\rho^{\mu }(dx)\mu_{x}(df),\;\text{on}\;{\mathbb{R}}^{d}\times ℱ,$$

with field of conditional probabilities:

(3) $$x\mapsto \mu_{x}\in {ℳ}_{P}(ℱ),$$

Dense tissue is modeled as μ having marginal $\rho^{\mu }(dx)=\rho (x)dx$ continuous with Lebesgue measure on ${\mathbb{R}}^{d}$:

(4) $$\mu (dx,df)=\rho (x)dx\mu_{x}(df).$$

A fundamental link between the molecular and continuum tissues can be addressed through the law of large numbers since if ${\left(x_{i},f_{i}\right)}_{i\geq 0}$ is an independent and identically distributed sample drawn from law μ/M of ${\mathbb{R}}^{d}\times ℱ$ where $M=\int_{{\mathbb{R}}^{d}\times ℱ}\mu (dx,df)$ is the total mass of such μ, then we have almost surely the weak convergence

(5) $$\mu_{N}:=\frac{M}{N}\sum_{i=1}^{N}\delta_{x_{i}}⊗\delta_{f_{i}}\to \mu .$$

Passing from the tissue scales to the molecular-cellular scales of Figure 1(a) behooves us to introduce a scale space so that empirical averages which govern it are repeatable. Figure 1(b) depicts our multiscale model of a brain as a sequence of measures:

(6) $$\mu ={\left(\mu^{\ell }\right)}_{\ell =0,1,\cdots }.$$

Our idealization of Brainspace as a sequence of measures is depicted in Figure 1 descending from the coarse tissue scale (top b) to the finest particle representation (bottom b), with color representing function f∈ℱ and radii space scale. Throughout, the range of scales is denoted shorthand ℓ≥0 to mean $0\leq \ell <\ell_{\max}$.

### 2.2. Nonlinear Transformation Model for Crossing Scales.

The brain being a multiscale collection of measures requires us to be able to transform from one scale to another. We do this by associating to each scale transformation a transition kernel acting on the measure at that scale. The transition kernels carry resolution scales σ or reciprocally bandwidths, analogous to Planck’s scale.

We introduce the abstract representation of our system as a collection of descriptive elements z∈𝓩 made from spatial and functional features. We transform our mathematical measurefi μ(⋅) on 𝓩 generating new measures $\mu^{\prime }(\cdot )$ on ${𝓩}^{\prime }$ defining correspondences via transition kernels $z\mapsto k\left(z,dz^{\prime }\right)$, with the kernel acting on the measures transforming as

(7) $$K[\mu ]\left(dz^{\prime }\right)=\int_{𝓩}k\left(z,dz^{\prime }\right)\mu (dz).$$

This implies the particles transformas $K\left[\delta_{z_{i}}\right]\left(dz^{\prime }\right)=k\left(z_{i},dz^{\prime }\right)$.

Figure 1(c) shows the cascade of operations, the first transforming linearly $\mu =\sum_{i\in I}w_{i}\delta_{x_{i}}⊗\delta_{f_{i}}\mapsto v$ on ${\mathbb{R}}^{d}\times ℱ$ according to $v(\cdot )=\int_{{\mathbb{R}}^{d}\times ℱ}k_{1}((x,f),\cdot )\mu (dx,df)$, and the second transforming nonlinearly $\nu \mapsto \mu^{\ell }$ on ${\mathbb{R}}^{d}\times {ℱ}^{\ell }$ smoothing at scale ℓ the conditional distribution on features:

(8) $$\{\begin{array}{l} T_{1}:\mu \mapsto v(\cdot )=\sum_{i\in I}w_{i}k_{1}\left(\left(x_{i},f_{i}\right),\cdot \right),\\ \;\text{with}\;v(dx,df)=\rho^{v}(dx)v_{x}(df),\\ T_{2}:v\mapsto \mu^{\ell }(\cdot )=\int_{{\mathbb{R}}^{d}}k_{2}\left(\left(x,v_{x}\right),\cdot \right)\rho^{v}(dx). \end{array}$$

Smooth space resampling projects particles to the continuum using a smooth resampling process defined by x↦π(x,y), the fraction particle x transfers to y, giving

(9a) $$k_{1}((x,f),\cdot )=\int_{{\mathbb{R}}^{d}}\pi (x,y)\delta_{y}⊗\delta_{f}(\cdot )dy,$$

$$v(dx,df)=w^{\ell }(x)dx\;v_{x}(df),$$

(9b) $$\text{with}\;\{\begin{array}{l} w^{\ell }(x)=\sum_{i\in I}w_{i}\pi \left(x_{i},x\right),\\ v_{x}=\sum_{i\in I}\frac{w_{i}}{w^{\ell }(x)}\pi \left(x_{i},x\right)\delta_{f_{i}}. \end{array}$$

Notice $\rho^{v}(dx)=w^{\ell }(x)dx$. Feature reduction uses maps from machine learning, $\alpha \mapsto \phi (\alpha )\in {ℱ}^{\ell }$, $\int_{ℱ}\alpha (df)=1$:

(9c) $$k_{2}((x,\alpha ),\cdot )=\delta_{x}⊗\delta_{\phi (\alpha )}(\cdot ),$$

(9d) $$\begin{array}{l} \mu^{\ell }(\cdot )=\int_{R^{d}}w^{\ell }(x)k_{2}\left(\left(x,v_{x}\right),\cdot \right)dx\\ \;=\int_{R^{d}}w^{\ell }(x)\delta_{x}⊗\delta_{\phi \left(v_{x}\right)}(\cdot )dx. \end{array}$$

For computing we resample to the computational lattices ${\left(Y_{j}^{\ell }\subset {\mathbb{R}}^{d},y_{j}\right)}_{j\in I_{\ell },\ell \geq 0}$ interpolating from the continuum to the lattice centers $y_{j}\in Y_{j}^{\ell }$; defining $\pi (x,Y):=\int_{Y}\pi (x,y)\;dy$, gives the transition kernel and transformed measure:

$$k_{1}((x,f),\cdot )=\sum_{j\in I_{\ell }}\pi \left(x,Y_{j}^{\ell }\right)\delta_{y_{j}}⊗\delta_{f}(\cdot ),$$

$$\mu^{\ell }(\cdot )=\sum_{j\in I_{\ell }}w_{j}^{\ell }k_{2}\left(\left(y_{j},v_{y_{j}}\right),\cdot \right)=\sum_{j\in I_{\ell }}w_{j}^{\ell }\delta_{y_{j}}⊗\delta_{\phi \left(v_{y_{j}}\right)}(\cdot ),$$

(10) $$\text{with}\;\{\begin{array}{l} w_{j}^{\ell }=\sum_{i\in I}w_{i}\pi \left(x_{i},Y_{j}^{\ell }\right),\\ v_{y_{j}}=\sum_{i\in I}\frac{w_{i}}{w_{j}^{\ell }}\pi \left(x_{i},Y_{j}^{\ell }\right)\delta_{f_{i}}. \end{array}$$

### 2.3. Dynamical Systems Model via Varifold Action of Multiscale Diffeomorphisms.

We want to measure and cluster brains by building a metric space structure. We do this by following the original program of D’Arcy Thompson building bijective correspondences. In this setting, this must be done at every scale with each scale having different numbers of particles and resolutions. We build correspondences between sample brains via dense connections of the discrete particles to the continuum at all scales using the diffeomorphism group and diffeomorphic transport. For this, define the group of k-times continuously differentiable diffeomorphisms $\varphi \in G_{k}$ with group operation function composition $\varphi \circ \varphi^{\prime }$. For any brain $\mu =\sum_{i\in I}w_{i}\delta_{x_{i}}⊗\delta_{f_{i}}\in ℳ\left({\mathbb{R}}^{d}\times ℱ\right)$, the diffeomorphisms act

(11a) $$(\varphi ,\mu )\mapsto \varphi \cdot \mu =\sum_{i\in I}w_{i}\left|d\varphi \left(x_{i}\right)\right|\delta_{\varphi \left(x_{i}\right)}⊗\delta_{f_{i}}.$$

The tissue has classical density $\mu =\int_{{\mathbb{R}}^{d}}\rho (x)\delta_{x}⊗\delta_{f(x)}dx$ with feature f(x) indexed over space, with action

(11b) $$\varphi \cdot \mu =\int_{{\mathbb{R}}^{d}}\rho (x)|d\varphi (x)|\delta_{\varphi (x)}⊗\delta_{f(x)}dx.$$

Space scales are represented with the group product, $\varphi :={\left(\varphi^{\ell }\right)}_{\ell \geq 0}$, acting component-wise with action

(11c) $$\varphi \cdot \mu :={\left(\varphi^{\ell }\cdot \mu^{\ell }\right)}_{\ell \geq 0}.$$

We call the |dφ(x)| term in the action the “copy and paste” varifold action. It enables the crucial property that when a tissue is extended to a larger area, the total number of its basic constituents increases accordingly with total integral not conserved, in contrast to classic measure or probability transport.

Dynamics occurs by generating the diffeomorphism as flows $t\mapsto \varphi_{t}:={\left(\varphi_{t}^{\ell }\right)}_{\ell \geq 0}$, with dynamics controlled by vector fields $t\mapsto u_{t}:={\left(u_{t}^{\ell }\right)}_{\ell \geq 0}$ via the ordinary differential equation at each scale satisfying

(12a) $${\dot{\varphi }}_{t}^{\ell }=u_{t}^{\ell }\circ \varphi_{t}^{\ell }$$

The controls are coupled by successive refinements $v^{\ell },\ell \geq 0$, with $u^{-1}=0$:

(12b) $$u_{t}^{\ell }=u_{t}^{\ell -1}+v_{t}^{\ell }.$$

**Figure 2:**
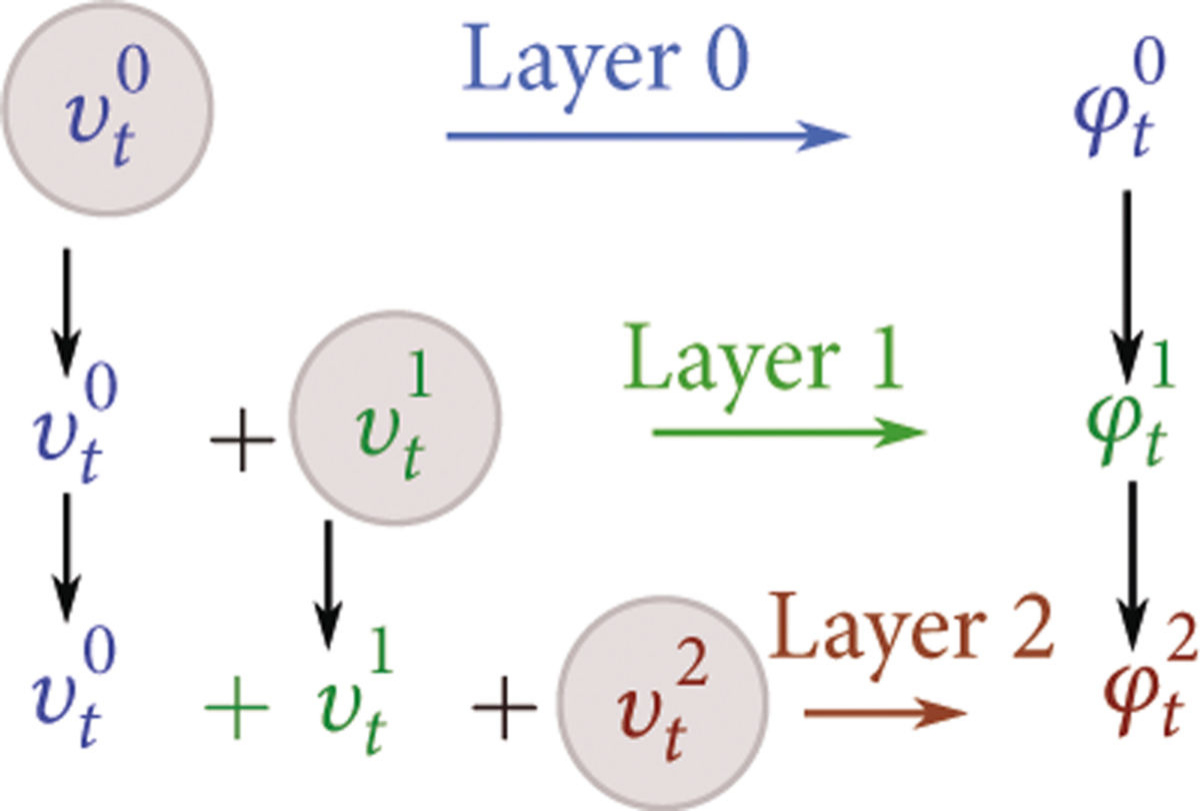
Hierarchical system, controls $u^{\ell }=u^{\ell -1}+v^{\ell },u^{0}=v^{0}$, and flows $\varphi^{\ell },\ell =0,1,\cdots$.

To control smoothness of the maps, we force the vector fields to be elements of reproducing kernel Hilbert spaces (RKHS’s) $V_{\ell }$, norms $∥\cdot {∥}_{V_{\ell }}$, with multiscale $u\in V:=\prod_{\ell \geq 0}V_{\ell }$. Each RKHS is taken to have a diagonal kernel with $g_{\ell }$ the Green’s function,

(13) $$k_{\ell }(\cdot ,\cdot )=g_{\ell }(\cdot ,\cdot )\text{id,}$$

where id is the d×d identity matrix; see [25] for nondiagonal kernels. Geodesic mapping flows under a control process along paths of minimum energy respecting boundary conditions. Figure 2 shows the multiscale control hierarchy.

The multiscale dynamical controls are written ${\dot{\varphi }}_{t}=u_{t}\circ \varphi_{t}:={\left(u_{t}^{\ell }\circ \varphi_{t}^{\ell }\right)}_{\ell \geq 0}$, with observer and dynamics equation:

(14) $$\{\begin{array}{ll} \varphi_{t}\cdot \mu ,t\in [0,1] & \text{observer,}\\ {\dot{\varphi }}_{t}=u_{t}\circ \varphi_{t},\varphi_{0}=\text{Id} & \text{dynamics}. \end{array}$$

Dynamics translates into a navigation in the orbit of brains and provides a metric distance between brains. Paths of minimum energy connecting the identity $\varphi_{0}=\text{Id}$ to any fixed boundary condition (BC) $\varphi_{1}$ where $\varphi_{1}$ is accessible defines the distance extending LDDMM [26] to a hierarchy of diffeomorphisms and is a geodesic for an associated Riemannian metric [25].

The metric from $\mu_{0}$ to $\mu_{1}$ in the orbit accessible from $\mu_{0}$ via diffeomorphisms is the shortest length geodesic paths with BCs $\varphi_{0}\cdot \mu_{0}=\mu_{0}$ and $\varphi_{1}\cdot \mu_{0}=\mu_{1}$. This extension to multiscale LDDMM equation (34) is given in Section 4.3 where we discuss the smoothness required for the geodesics to define a metric and specify the optimal control problem in the state equation (36).

### 2.4. Geodesic Brain Mapping via the Varifold Measure Norm.

The BC matching brains is defined using measure norms with equality meaning brains are equal, with small normed difference meaning brains are similar; for particle brains, $\mu =\sum_{i\in I}w_{i}\delta_{x_{i}}⊗\delta_{f_{i}}$; for tissue, $\mu =\int_{{\mathbb{R}}^{d}}\delta_{x}⊗\mu_{x}\rho (x)dx$. Geodesic mapping controls the flow to minimize energy simultaneously minimizing the norm distance to the target. Every brain has a variable number of particles with no correspondence between particles. Varifold measure norms accommodate these variabilities. The varifold norm is constructed modeling the particles as elements $\delta_{x}⊗\delta_{f}\in W^{*}$ of the dual space of an RKHS *W* associated with the isometry $K_{W}:W^{*}\to W$ and kernel function $k_{W}$ defining the inner product. We introduce the dual bracket notation for h∈W, $\mu \in W^{*}$, and for μ a measure, $\left(\mu |h):=\int_{{\mathbb{R}}^{d}\times ℱ}h(x,f)\mu (dx,df\right)$. Then, we have

(15a) $$\begin{array}{l} {\langle \delta_{x_{i}}⊗\delta_{f_{i}},\delta_{x_{j}}⊗\delta_{f_{j}}\rangle }_{W^{*}}=\left(\delta_{x_{i}}⊗\delta_{f_{i}}|K_{W}\left[\delta_{x_{j}}⊗\delta_{f_{j}}\right]\right)\\ \;=k_{W}\left(\left(x_{i},f_{i}\right),\left(x_{j},f_{j}\right)\right). \end{array}$$

The norm-square for particle and tissue measures reduces to

(15b) $$∥\mu {∥}_{W^{*}}^{2}:=\left(\mu |K_{W}[\mu ]\right)=\int_{{\mathbb{R}}^{d}\times ℱ}K_{W}[\mu ](x,f)\mu (dx,df),$$

(15c) $$=\{\begin{array}{l} \sum_{i,j\in I}w_{i}w_{j}k_{W}\left(\left(x_{i},f_{i}\right),\left(x_{j},f_{j}\right)\right)\\ \int_{{\mathbb{R}}^{d}}\int_{{\mathbb{R}}^{d}}\rho (x)\rho (y)k_{W}\left((x,f),\left(y,f^{\prime }\right)\right)\mu_{x}(df)\mu_{y}\left(df^{\prime }\right)dxdy. \end{array}$$

The hierarchical norms across the scales become $∥\mu {∥}_{W^{*}}^{2}:=\sum_{\ell \geq 0}{\left\|\mu^{\ell }\right\|}_{W_{\ell }^{*}}^{2}$.

The optimal control ${\left(u_{t}\right)}_{0\leq t\leq 1}$ is square-integrable for the *V*-norms for particles and tissue, satisfying for α>0:

(16) $$\underset{\begin{array}{c} {\left(u_{t}\right)}_{0\leq t\leq 1}\in L^{2}([0,1],V)\\ {\dot{\varphi }}_{t}=u_{t^{\circ }}\varphi_{t},\varphi_{0}=\mathrm{Id} \end{array}}{\min}\frac{1}{2}\sum_{\ell \geq 0}\int_{0}^{1}{\left\|u_{t}^{\ell }-u_{t}^{\ell -1}\right\|}_{V_{\ell }}^{2}dt+\frac{\alpha }{2}{\left\|\varphi_{1}\cdot \mu -\mu_{\text{obs}}\right\|}_{W^{*}}^{2}.$$

The control flows the measures $\mu \left(q_{t}\right):=\varphi_{t}\cdot \mu$ with state: processes $t\mapsto q_{t}$ and endpoint $U\left(q_{1}\right)=\sum_{\ell \geq 0}U\left(q_{1}^{\ell }\right)$

(17a) $$q_{t}:=\{\begin{array}{ll} {\left(x_{i,t}=\varphi_{t}\left(x_{i}\right),w_{i,t}=w_{i}\left|d\varphi_{t}\right|\left(x_{i}\right)\right)}_{i\in I} & \text{particle,}\\ {\left(\varphi_{t}(x),w_{t}(x)=w(x)\left|d\varphi_{t}\right|(x)\right)}_{x\in {\mathbb{R}}^{d}} & \text{tissue,} \end{array}$$

(17b) $$U\left(q_{1}^{\ell }\right):=\frac{\alpha }{2}{\left\|\mu \left(q_{1}^{\ell }\right)-\mu_{\text{obs}}^{\ell }\right\|}_{W_{\ell }^{*}}^{2}=\frac{\alpha }{2}{\left\|\varphi_{1}^{\ell }\cdot \mu^{\ell }-\mu_{\text{obs}}^{\ell }\right\|}_{W_{\ell }^{*}}^{2}.$$

The endpoint $q_{1}\mapsto U\left(q_{1}\right)$ is modeled as continuously differentiable in the states.

Hamiltonian control of particles reparameterizes (16) in momentum “costates” $t\mapsto p_{t}={\left(p_{i,t}^{x},p_{i,t}^{w}\right)}_{i\in I}$, with momentum at each scale a function of same scale dynamics (ℓ index suppressed). The optimal control averages Green’s functions (13) across scales ${\bar{g}}_{\ell }:=\sum_{j=0}^{\ell }g_{j}$ with $\nabla_{1}\bar{g}(a,b)$ the gradient on the first variable *a*; define $\varphi_{t,s}=\varphi_{s}\circ \varphi_{t}^{-1}$; then

(18a) $$u_{t}^{\ell }(\cdot )=\sum_{j\geq 0}\sum_{i\in I_{j}}\left({\bar{g}}_{\ell \wedge j}\left(x_{i,t}^{j},\cdot \right)p_{i,t}^{x,j}+p_{i,t}^{w,j}w_{i,t}^{j}\nabla_{1}{\bar{g}}_{\ell \wedge j}\left(x_{i,t}^{j},\cdot \right)\right),$$

(18b) $$\{\begin{array}{l} p_{i,t}^{x}={\left(d\varphi_{t,1}\right)}^{T}\left(x_{i,t}\right)p_{i,1}^{x}+p_{i,1}^{w}w_{i,1}\int_{t}^{1}{\left(d\varphi_{t,s}\right)}^{T}\left(x_{i,t}\right)\nabla \left(\text{div}\;u_{s}\right)\left(x_{i,s}\right)ds,\\ p_{i,t}^{w}=p_{i,1}^{w}\left|d\varphi_{t,1}\right|\left(x_{i,t}\right). \end{array}$$

Control of tissue reparameterizes (16) in $t\mapsto p_{t}={\left(p_{t}^{\varphi }(x),p_{t}^{w}(x)\right)}_{x\in {\mathbb{R}}^{d}}$; if $\left(p_{1}^{\varphi },p_{1}^{w}\right)$ is absolutely continuous, it remains for t<1:

(19a) $$\begin{array}{l} u_{t}^{\ell }(\cdot )=\sum_{j\geq 0}\int_{{\mathbb{R}}^{d}}({\bar{g}}_{\ell \wedge j}\left(\varphi_{t}^{j}(x),\cdot \right)p_{t}^{\varphi ,j}(x)\\ \;+p_{t}^{w,j}(x)w_{t}^{j}(x)\nabla_{1}{\bar{g}}_{\ell \wedge j}\left(\varphi_{t}^{j}(x),\cdot \right))dx, \end{array}$$

(19b) $$\{\begin{array}{l} p_{t}^{\varphi }={\left(d\varphi_{t,1}\right)}^{T}\circ \varphi_{t}p_{1}^{\varphi }+p_{1}^{w}w_{1}\int_{t}^{1}{\left(d\varphi_{t,s}\right)}^{T}\circ \varphi_{t}\nabla \left(\text{div}\;u_{s}\right)\circ \varphi_{s}ds,\\ p_{t}^{w}=p_{1}^{w}\left|d\varphi_{t,1}\right|\circ \varphi_{t}. \end{array}$$

The endpoint momentum for particles ${\left(p_{i,1}^{x},p_{i,1}^{w}\right)}_{i\in I}$ and dense tissue ${\left(p_{1}^{\varphi }(x),p_{1}^{w}(x)\right)}_{x\in {\mathbb{R}}^{d}}$ is given by the variation of the norm-square match determined by $h_{q_{1}}$, the kernel smoothing of the difference of the measures:

(20a) $$h_{q_{1}}:=\alpha K_{W}\left[\mu \left(q_{1}\right)-\mu_{\text{obs}}\right]\;\text{on}\;{\mathbb{R}}^{d}\times ℱ\text{,}$$

with endpoint momentum

(20b) $$\{\begin{array}{l} p_{i,1}^{x}=-\nabla_{x_{i}}U\left(q_{1}\right)=-w_{i,1}\nabla_{x_{i}}h_{q_{1}}\left(x_{i,1},f_{i}\right),\\ p_{i,1}^{w}=-\nabla_{w_{i}}U\left(q_{1}\right)=-h_{q_{1}}\left(x_{i,1},f_{i}\right), \end{array}$$

(20c) $$\{\begin{array}{l} p_{1}^{\varphi }(x)=-\nabla_{\varphi (x)}U\left(q_{1}\right)=-w_{1}(x)\rho (x)\int_{ℱ}\nabla_{\varphi }h_{q_{1}}\left(\varphi_{1}(x),f\right)\mu_{x}(df),\\ p_{1}^{w}(x)=-\nabla_{w(x)}U\left(q_{1}\right)=-\rho (x)\int_{ℱ}h_{q_{1}}\left(\varphi_{1}(x),f\right)\mu_{x}(df). \end{array}$$

The RKHS kernel defined in the Section 4.2, Eqn. (29) is a separable Gaussian in space and function. The optimal control at any scale “averages” all the particle/tissue data across scales. Section 4.4 establishes the smoothness for the Hamiltonian equations. Section 4.5 establishes the variation and smoothness for the norm gradients.

We emphasize that the varifold action gives the continuum problem unifying with image-based LDDMM [26] such as studied by the MRI community. Taking $I(y)\in {\mathbb{R}}^{+}$ with $\mu_{y}=\delta_{I(y)}$, $\mu :=\int_{{\mathbb{R}}^{d}}\delta_{y}⊗\delta_{I(y)}dy$ the action becomes

(21) $$\varphi_{t}\cdot \mu =\int_{{\mathbb{R}}^{d}}\left|d\varphi_{t}(y)\right|\delta_{\varphi_{t}(y)}⊗\delta_{I(y)}dy=\int_{{\mathbb{R}}^{d}}\delta_{x}⊗\delta_{I\circ \varphi_{t}^{-1}(x)}dx.$$

### 2.5. MRI and Digital Pathology for Tau Histology in Alzheimer’s

#### 2.5.1. Bayes Segmentation of MRI.

**Figure 3:**
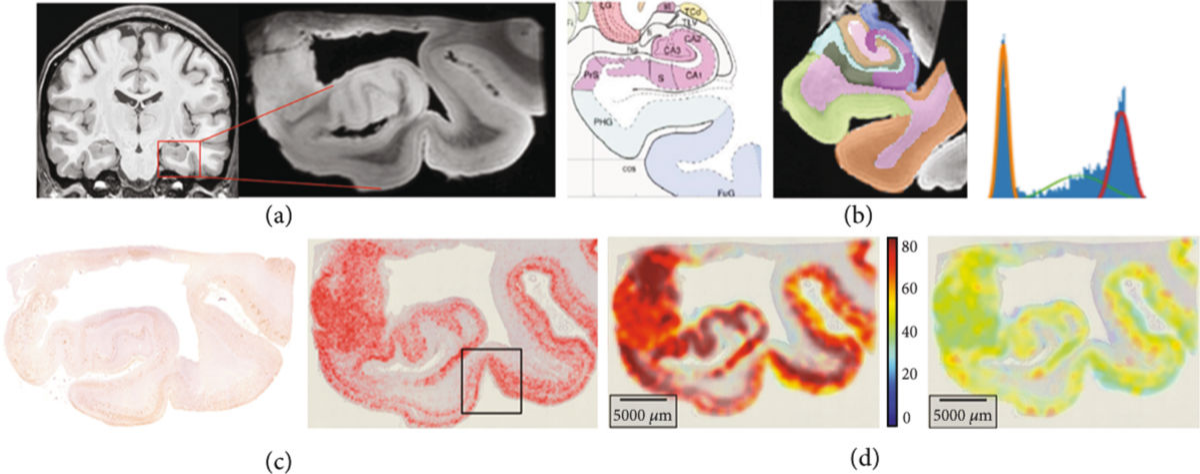
(a, b) Medial temporal lobe at 1 mm and high-field 200 *μ*m MRI; Mai-Paxinos atlas section of the MRI with the hippocampus and entorhinal cortex; (b) shows the Mai Paxinos section and Bayes compartments. (c, d) Alzheimer 4 *μ*m tau histology (red) from section depicted via high-field MRI (a, b); (c) shows detected tau particles in two sections. The box depicts transentorhinal region from (a, b). (d) Shows the mean particle size and standard deviation at micrometer tissue scales; deep red color denotes 80 *μ*m^2^ tau area.

Figure 3 shows the multiscale data from the clinical BIOCARD study [6] of Alzheimer’s disease within the medial temporal lobe [7, 8, 27]. Figure 3(a) shows the clinical magnetic resonance imaging (MRI) with the high-field 200μm MRI scale (b) shown depicting the medial temporal lobe including the collateral sulcus and lateral bank of the entorhinal cortex. Bayes classifiers for brain parcellation performs feature reduction as a key step for segmentation at tissue scales [28]. Feature reduction maps the distribution on gray levels ℱ=[0,255] to probabilities on *N* tissue types, defined by the integration over the decision regions $\theta_{n}\subset [0,255]$:

(22) $$\phi_{n}\left(\mu_{x}\right)=\int_{\theta_{n}}\mu_{x}(df)=p_{n},\;n=1,\cdots ,N.$$

Figure 3(b) depicts a Bayes classifier for gray, white, and cerebrospinal fluid compartments generated from the temporal lobe high-field MRI section corresponding to the Mai-Paxinos section (panel 3, top row).

#### 2.5.2. Gaussian Scale-Space Resampling of Tau Histology.

For histology at the molecular scales, the measure encodes the detected tau and amyloid particles $\mu =\sum_{i\in I}w_{i}\delta_{x_{i}}⊗\delta_{f_{i}}$ for fine-scale particles with function the size $ℱ={\mathbb{R}}^{+}$. Figure 3(c) shows the detected tau particles as red dots at 4 *μ*m. We use computational lattices to interpolate between scales reapportioning to the lattice centers ${\left(y_{j}\in Y_{j}\subset {\mathbb{R}}^{d}\right)}_{j\in I_{\ell }}$ via Gaussian resampling $x\mapsto \pi_{\sigma }\left(x,Y_{j}\right)$. Feature reduction to the tissue scales maps to the first two moments (Figure 3(d)) of mean and variance of particle size ${ℱ}^{\ell }\subset {\mathbb{R}}^{2}$ :

(23a) $$\pi_{\sigma }\left(x_{i},Y_{j}\right)=\frac{1}{\left(2\pi \sigma^{2}\right)}\int_{Y_{j}}e^{-{\left|y-x_{i}\right|}^{2}/2\sigma^{2}}dy,$$

(23b) $$\begin{array}{l} \mu^{\ell }=\sum_{j\in I_{\ell }}w_{j}^{\ell }\delta_{y_{j}}⊗\delta_{\phi \left(v_{y_{j}}\right)},\\ \;\text{with}\;\{\begin{array}{l} w_{j}^{\ell }=\sum_{i\in I}w_{i}\pi_{\sigma }\left(x_{i},Y_{j}\right),\\ v_{y_{j}}=\frac{1}{w_{j}^{\ell }}\sum_{i\in I}w_{i}\pi_{\sigma }\left(x_{i},Y_{j}\right)\delta_{f_{i}},\\ \phi \left(v_{y_{j}}\right)=\left(\int_{ℱ}fv_{y_{j}}(df),\int_{ℱ}f^{2}v_{y_{j}}(df)\right). \end{array} \end{array}$$

The millimeter tissue scale depicts the global folding property of the tissue. The color codes the mean tau particle area as a function of position at the tissue scales with deep red denoting 80 *μ*m^2^ maximum tau area for the detected particles.

### 2.6. Cellular Neurophysiology: Neural Network Temporal Models.

Single-unit neurophysiology uses temporal models of spiking neurons with a “neural network” $\mu =\sum_{i}\delta_{x_{i}}⊗\delta_{f_{i}}$, $x_{i}\in {\mathbb{R}}^{d}$, $f_{i}\in ℱ$ taking each neuron $x_{i}$ modeled as a counting measure in time $N_{i}(t),t\geq 0$ with the spike times the feature $f_{i}={\left(t_{k}\right)}_{1\leq k\leq n_{f_{i}}}$:

(24) $$ℱ=\left\{f={\left(t_{k}\right)}_{1\leq k\leq n_{f}}:n_{f}\geq 1,t_{1}<t_{2}\cdots <t_{n_{f}}\right\}.$$

The Poisson model with intensity $\lambda (t),t\geq t_{0}$ [10] has probabilities $\mathrm{Pr}\left(N_{i}(t)=n\right)=\left({\left(\int_{t_{0}}^{t}\lambda (s)ds\right)}^{n}/n!\right)e^{-\int_{t_{0}}^{t}\lambda (s)ds}$.

Post-stimulus time (PST) [29] and interval histograms are used to examine the instantaneous discharge rates and interspike interval statistics [30]. The interval histogram abandons the requirement of maintaining the absolute phase of the signal for measuring temporal periodicity and phase locking. Synchrony in the PST is measured using binning $\left[b_{i},b_{i+1}\right),i=1,\cdots ,B$ and Fourier transforms, $j=\sqrt{-1}$:

(25) $$\phi_{n}^{\text{PST}}\left(\mu_{x}\right)=\sum_{i}e^{j\omega_{n}i}\int_{ℱ}\frac{1}{n_{f}}\sum_{k=1}^{n_{f}}1_{\left[b_{i},b_{i+1}\right)}\left(t_{k}\right)\mu_{x}(df),\;n=0,1\cdots .$$

The n=0 frequency computes integrated rate; each phase-locked feature is complex $\phi_{n}\in \mathbb{C}$.

### 2.7. Scale-Space Resampling of RNA to Cell and Tissue Scales.

**Figure 4:**
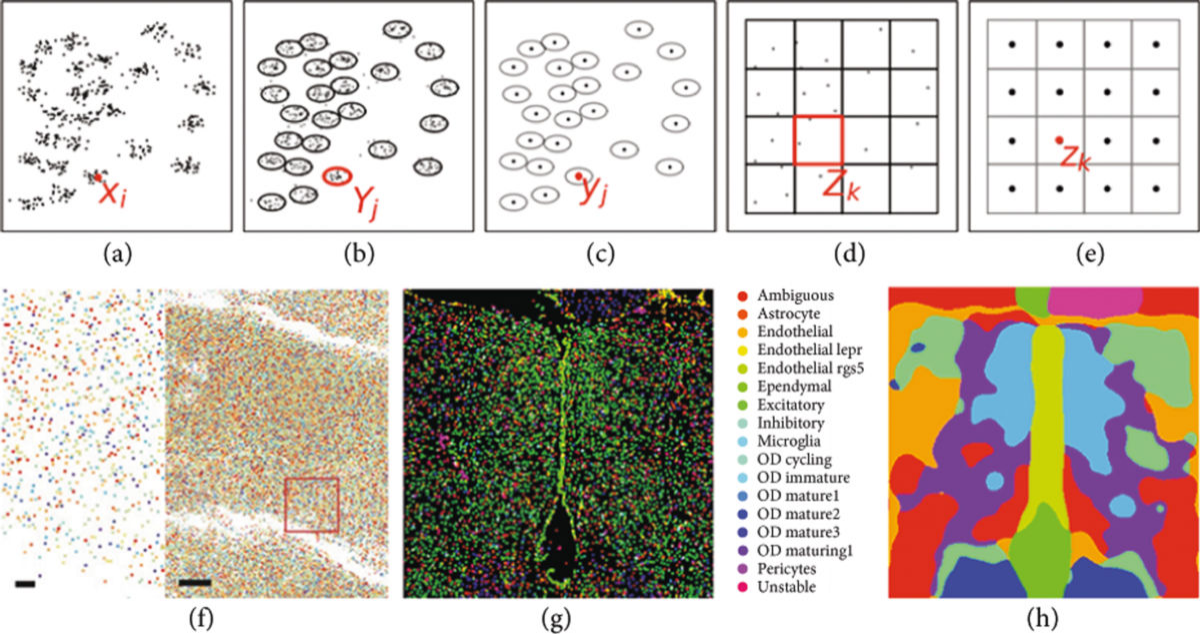
(a–e) Show the cartoon of multiscale renormalization of particles to cells and cell centers to regular lattice representing the tissue. (f) Shows RNA marks from [35] of the 167 RNA gene species (bar scales 1, 10 *μ*m); (g, h) shows data from [34] showing 17 cell types clustered on cell centers (g); (h) shows *K* = 10 means clustering to tissue with Gaussian resampling with *σ* = 25 pix on the 450 × 200 pix^2^ grid.

Methods in spatial transcriptomics which have emerged for localizing and identifying cell types via marker genes and across different cellular resolutions [4, 31–35] present the opportunity of localizing in spatial coordinates the transcriptionally distinct cell types. Depicted in Figure 4 are the molecular measurements at the micron scales with MERFISH [34] at three different scales.

The molecular measures represent RNA locations ${\left(x_{i}\in {\mathbb{R}}^{2}\right)}_{i\in I_{R}}$ with sparse RNA features $\mu^{R}=\sum_{i\in I_{R}}\delta_{x_{i}}⊗\delta_{f_{i}}$, ${ℱ}^{R}={\mathbb{R}}^{167}$. Crossing to cells resamples to their centers ${\left(y_{j}\in Y_{j}\subset {\mathbb{R}}^{2}\right)}_{j\in I_{C}}$ partitioning into the closest subsets as defined by the distance $d\left(x_{i},Y_{j}\right)$ of particle $x_{i}$ to cell $Y_{j}$, accumulating the mixtures of RNA within the closest cell. The cell scale feature is the conditional probability of the 17 cell type in ${ℱ}^{C}\subset \left[0,1\right]$^17^:

(26a) $$\pi \left(x_{i},Y_{j}\right)=\{\begin{array}{ll} 1 & \text{for}\;d\left(x_{i},Y_{j}\right)<d\left(x_{i},Y_{j^{\prime }\neq j}\right),\\ 0 & \text{otherwise,} \end{array}$$

$$\mu^{C}=\sum_{j\in I_{C}}w_{j}^{C}\delta_{y_{j}}⊗\delta_{\phi^{C}\left(v_{y_{j}}^{C}\right)},$$

(26b) $$\text{with}\;\{\begin{array}{l} w_{j}^{C}=\sum_{i\in I_{R}}\pi \left(x_{i},Y_{j}\right),\\ v_{y_{j}}^{C}=\frac{1}{w_{j}^{C}}\sum_{i\in I_{C}}\pi \left(x_{i},Y_{j}\right)\delta_{f_{i}},\\ \phi^{C}\left(v_{y_{j}}^{C}\right)={\left(\mathrm{Pr}\left\{C_{y_{j}}=c|v_{y_{j}}^{C}\right\}\right)}_{c=1,\cdots ,17}. \end{array}$$

For this example, the conditional probabilities on the RNA feature vector were found using principle components followed by Gaussian mixture modeling on $v_{y_{j}}^{C}$, onto c=1,⋯,17 following [34].

Resampling to the tissue lattice ${\left(z_{k}\in Z_{k}\subset {\mathbb{R}}^{2}\right)}_{k\in I_{T}}$ uses Gaussian rescaling with the new feature vector the probability of the cell at any position being one of 10 tissue lated using 10-means clustering on the cell type probabil-types ${ℱ}^{T}\subset {\left[0,1\right]}^{10}$. The probability of tissue type is calcuities. The distance for 10-means clustering is computed using the Fisher-Rao metric [36] between the feature laws $v_{z_{k}}^{T}$, partitioning the cell type feature space into 10 regions $\cup_{1⩽t⩽10}{ℱ}_{t}={ℱ}^{C}$ giving probability features:

(27a) $$\pi_{\sigma }\left(y_{j},Z_{k}\right)=\frac{1}{2\pi \sigma^{2}}\int_{Z_{k}}e^{-{\left|z-y_{j}\right|}^{2}/2\sigma^{2}}dz,$$

$$\mu^{T}=\sum_{k\in I_{T}}w_{k}^{T}\delta_{z_{k}}⊗\delta_{\phi^{T}\left(v_{z_{k}}^{T}\right)},$$

(27b) $$\text{with}\;\{\begin{array}{l} w_{k}^{T}=\sum_{j\in I_{C}}w_{j}^{C}\pi_{\sigma }\left(y_{j},Z_{k}\right),\\ v_{z_{k}}^{T}=\frac{1}{w_{k}^{T}}\sum_{j\in I_{C}}w_{j}^{C}\pi_{\sigma }\left(y_{j},Z_{k}\right)\delta_{f_{j}^{C}},\\ \phi^{T}\left(v_{z_{k}}^{T}\right)={\left(\mathrm{Pr}\left\{T_{z_{k}}=t|v_{z_{k}}^{T}\right\}\right)}_{t=1,\cdots ,10}. \end{array}$$

Figures 4(f)-4(h) show the RNA forming $\mu^{R}$ depicted as colored markers corresponding to the different gene species (bar scale 1, 10 microns). Figure 4(g) shows the feature space of 17 cell types making up $\mu^{C}$ associated to the maximal probability in the PCA projection from a classifier based on the mixtures of RNA at each cell location. Figure 4(h) shows the 10 tissue features of the 10-means procedure. In both scales, probabilities are concentrated on single classes via indicator functions computed on the conditional probabilities.

### 2.8. Geodesic Mapping for Spatial Transcriptomics, Histology, and MRI

#### 2.8.1. Navigation between Sections of Cell Identity in Spatial Transcriptomics.

**Figure 5:**
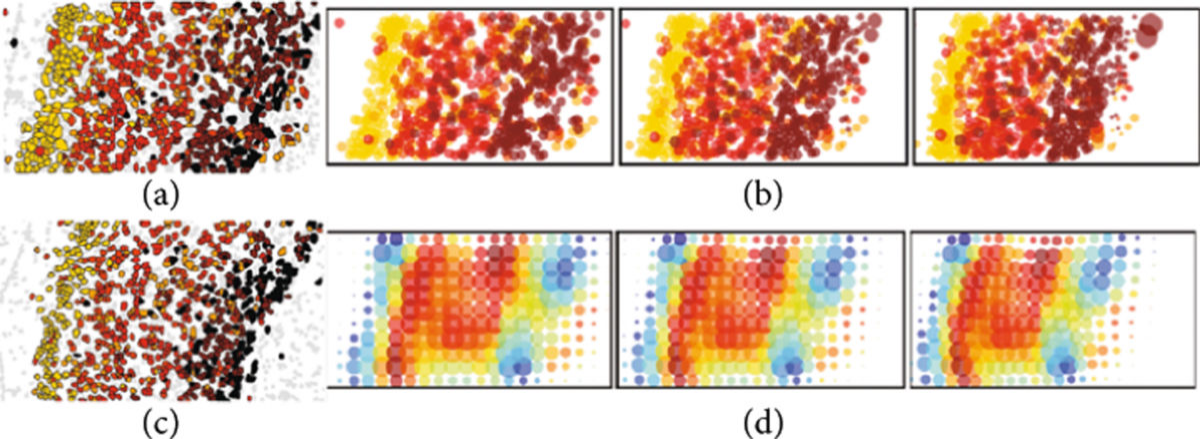
(a, c) Show neuronal cell types [4] for two sections: top template and bottom target. (b, d) Show $\varphi_{t}\cdot \mu$ for *t* = 0, 0:5, 1:0 of neuronal cell mapping cell types (b) and entropy (d). The varifold norm $\sigma_{x}^{\ell }=\sigma_{f}^{\ell }=10$ pix and 50 pix with the feature a one hot encoding of cell type. The vector fields have RKHS norm induced by the differential operator $L_{\ell }:={\left(\left(1-{\left(\alpha^{\ell }\right)}^{2}\nabla^{2}\right)\text{id}\right)}^{2},\alpha^{\ell }=5$ pix and 50 pix with full width at half maximum (FWHM) of the Green kernel FWHM = 20 pix and 145 pix, for ℓ=1,2, respectively.

Figures 5(a) and 5(b) show sections (a) from [4] depicting neuronal cell types via colors including excitatory cells eL2/3 (yellow), eL4 (orange), red eL5 (red), inhibitory cells ST (green), and VIP (light blue), each classified via high-dimensional gene expression feature vectors via spatial transcriptomics.

The measure on cell types $\mu^{C}=\sum_{i}w_{i}\delta_{x_{i}}⊗\delta_{f_{i}}$, ${ℱ}^{C}=\{\text{cell types}\}$ crosses to atlas tissue scales using $\pi_{\sigma }$ in ${\mathbb{R}}^{2}$ of equation (27a) with feature reduction expectations of moments, ${ℱ}^{T}=\{\text{size, mean}-\text{square, entropy}\}$:

(28) $$\mu^{T}=\sum_{j\in J}w_{j}^{T}\delta_{y_{j}}⊗\delta_{\phi \left(v_{y_{j}}\right)},$$

with $\phi \left(v_{y_{j}}\right)=\left(\sum_{{ℱ}^{c}}fv_{y_{j}}(f),\sum_{{ℱ}^{c}}f^{2}v_{y_{j}}(f),-\sum_{{ℱ}^{C}}v_{y_{j}}(f)\log v_{y_{j}}(f)\right)$.

Figures 5(b) and 5(d) show the tissue scale features associated to the cell type and the entropy. They shows the results of transforming the neuronal cells depicting the cell type (b) and entropy feature (d). The entropy is a measure of dispersion across the cell types given by the expectation of the log probability function with zero entropy meaning the space location feature distribution ν has all its mass on 1 cell type. Geodesic mapping enforces vector field smoothness via differential operators specifying the norms in the RKHS $∥v{∥}_{V_{\ell }}^{2}:=\left(L_{\ell }v|v\right)$ with $L_{\ell }:={\left(\left(1-{\left(\alpha^{\ell }\right)}^{2}\nabla^{2}\right)\text{id}\right)}^{2}$.

#### 2.8.2. Navigation between Sections of Histology.

**Figure 6:**
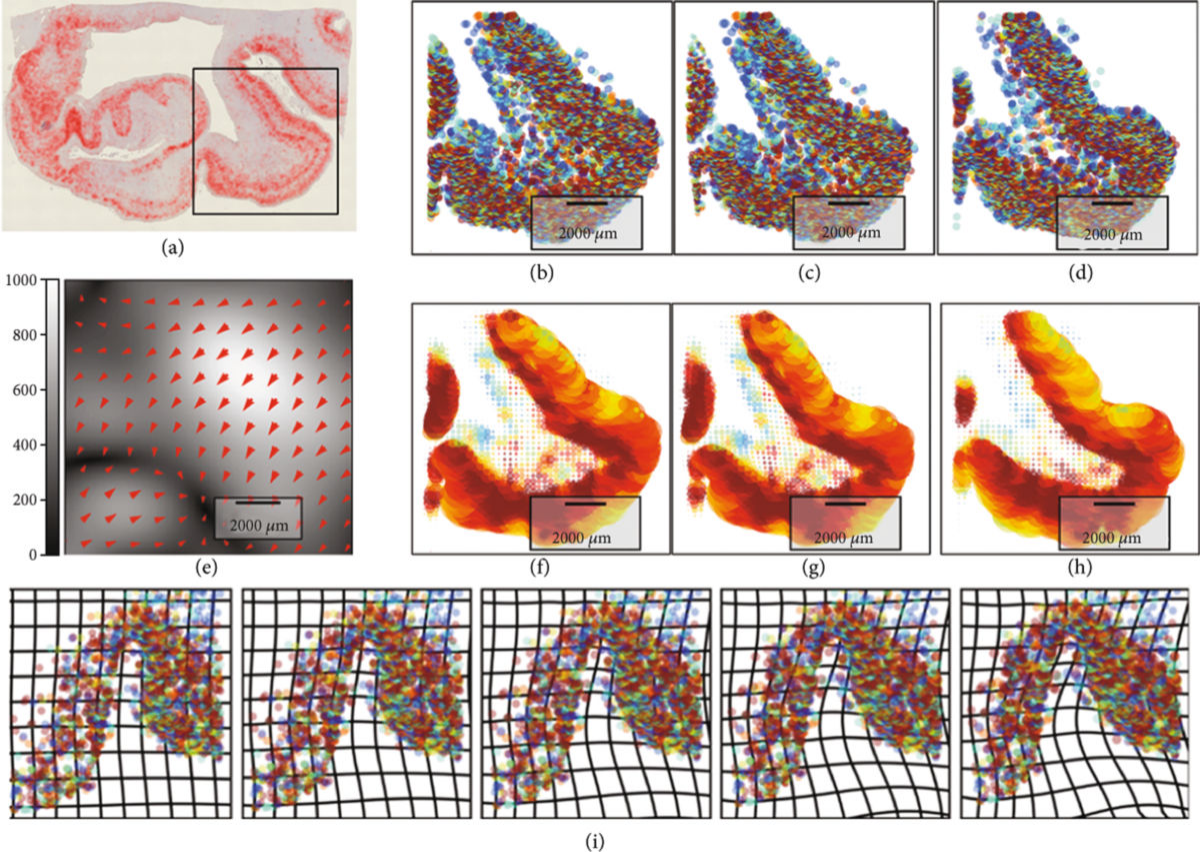
(a–h) Second section 4 *μ*m histology (similar to Figure 3) with the box depicting perirhinal cortex. (b–d) and (f–h) show template, mapped template, and target at the molecular (b–d) and tissue scales (f–h) showing the first moments of tau size on the perirhinal cortex; saturated red color denotes 80 *μ*m^2^ tau area. Mapped template shows narrowing 1000 *μ*m of perirhinal sulcus. (e) Depicts vector field encoding of the geodesic mapping with associated scale bar. (i) Geodesic navigation $\varphi_{t}\cdot \mu$ of collateral sulcus (Figure 3, box) showing 1000 *μ*m widening of folds for molecular and tissue scales depicting the mean transformation. The vector field mappings have RKHS norm induced by the differential operators with $L_{\ell }:={\left(\left(1-{\left(\alpha^{\ell }\right)}^{2}\nabla^{2}\right)\text{id}\right)}^{2}$ with $\alpha^{\ell }$ giving FWHM = 580 *μ*m and 3300 *μ*m, for ℓ=1,2, respectively; the varifold norm has $\sigma_{x}^{\ell }=800\;\mu \text{m}$ and 160 *μ*m.

Figures 6(a)–6(h) show navigation between the cortical folds of the 4*μ*m histology. Shown in (a) is a section showing the machine learning detection of the Tau particles. Figures 6(b)–6(d) and 6(f)–6(h) depict the template, mapped template, and target showing the mathematical measure representation of the perirhinal cortex constructed from the positions and sizes at the 4*μ*m scale (b–d) and reconstruction using Gaussian resampling onto the tissue scale (f–h). The color codes the mean of *μ_x_* representing tau area as a function of position at the tissue scales with deep red maximum denoting 80*μ*m^2^ of tau tangle area. The gradients in tau tangle area between superficial and deep layers are apparent with the deep red as high as 80 *μ*m^2^ for the innermost tissue fold. Panel (e) shows the vector field encoding of the geodesic transformation between the sections depicted by the bottom row transformation of grids. The narrowing of the banks of the perirhinal cortex is exhibited at the tissue scale for motion order 1000 *μ*m (brightness on scale bar).

Figure 6(i) shows the collateral sulcus fold at the boundary of the transentorhinal cortex region transforming based on the normed distances between sections with deformation motions 1000 *μ*m in size. Shown is the micron scale depicting the transformation of the gyrus with the color representing the entropy of the particle identity distribution.

#### 2.8.3. Mapping Digital Pathology from Histology to MRI Scales.

**Figure 7:**
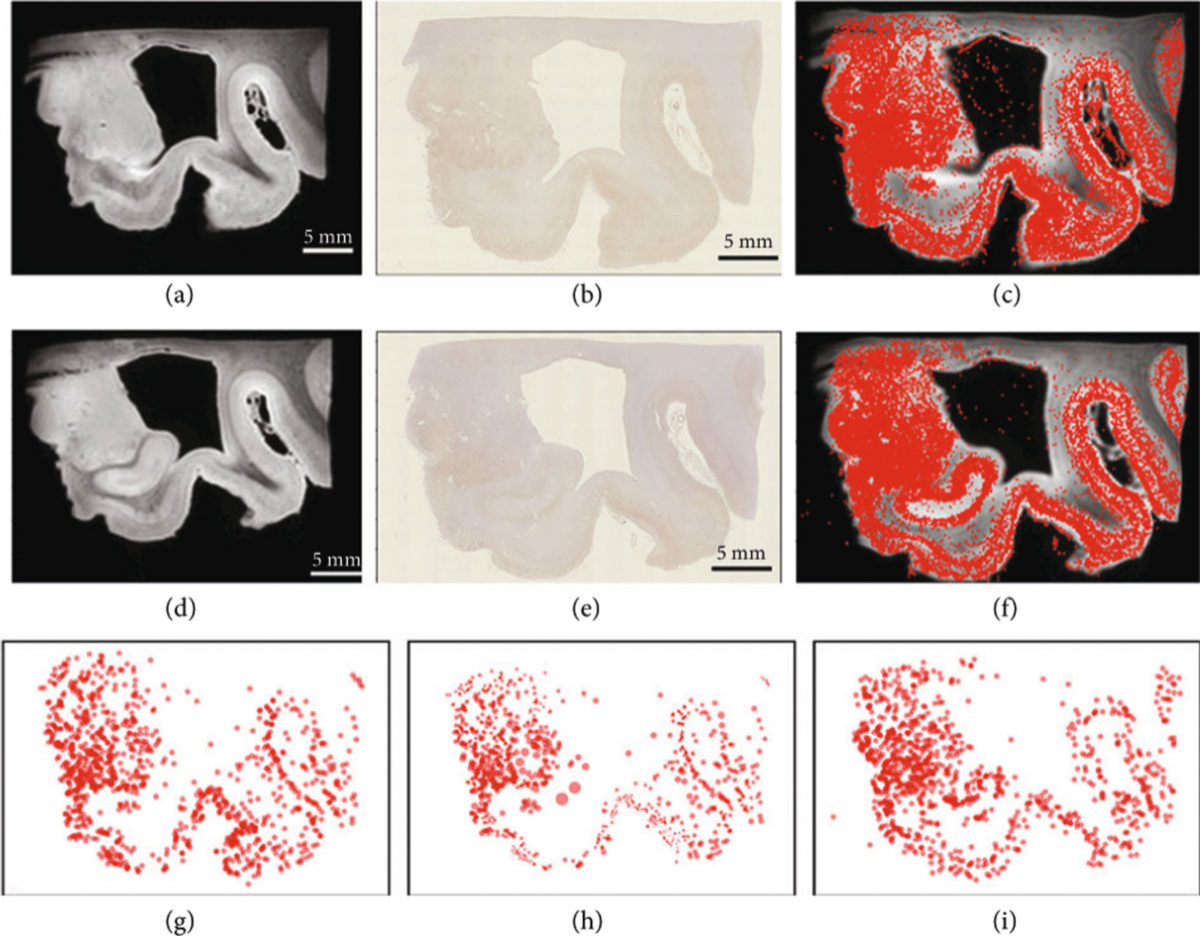
Whole brain section showing mapping MRI and histology at the multiple scales. (a–f) Show the MRI and tau histology for two sections with the detected tau particle superimposed over the MRI (c, f); (g–i) show the finest scales for the tau particles in the template and (h) the template tau particles mapped, with (i) showing the target tau particles; the varifold norm has $\sigma_{x}^{\ell }=4000\mu \text{m}$ and 100 *μ*m. The vector field mappings have RKHS norm induced by the differential operator $L_{\ell }:={\left(\left(1-{\left(\alpha^{\ell }\right)}^{2}\nabla^{2}\right)\text{id}\right)}^{2}$, $\alpha^{\ell }$ giving FWHM = 1500 *μ*m and 6400 *μ*m, for ℓ=1,2, respectively.

All of the examples thus far have created the multiscale data generated using the resampling kernels from the finest scales. As illustrated in our early figures, much of the data is inherently multiscale, with the measurement technologies generating the coarse scale representations. Shown in Figure 7 is data illustrating our Alzheimer’s study of postmortem MR images that are simultaneously collected with amyloid and tau pathology sections. MR images have a resolution of approximately 100*μ*m, while pathology images have a resolution of approximately 1*μ*m. For computational purposes, the MRI template and target images were downsampled to 759 and 693 particles, respectively, with the tau tangles downsampled to 1038 and 1028 particles, respectively. We treated every pixel in the MR image as a coarse scale particle with image intensity as its feature value equation (21) and every detected tau tangle as a fine-scale particle with a constant feature value and performed varifold matching to align to neighboring sections. The endpoint representing the two scales is $U=(1/2)\sum_{\ell =1,2}{\left\|\mu_{\text{temp}}^{\ell }-\mu_{\text{obs}}^{\ell }\right\|}_{W_{\ell }^{*}}^{2}$. For each scale norm, we use a varifold kernel given by the products of Gaussian distributions with the varifold measure norm equation (29), (30) at each scale. For the MRI scale, the weights are identical w=1 with the function component given by the MRI image value; for the tau particles, there is no function component making the kernel of equation (15) for all function values *f* in the varifold norm identically 1.

Figures 7(a)–7(f) show the imaging data for both sections. Figures 7(g)–7(i) show the transformed template image at the fine scale. The high-resolution mapping carries the kernels across all the scales as indicated by the geodesic equation for the control (18a). Notice the global motions of the high resolution of the fine particles.

## 3. Discussion

Computational anatomy was originally formulated as a mathematical orbit model for representing medical images at the tissue scales. The orbit model generalizes linear algebra to the group action on images by the diffeomorphism group. The orbit inherits a metric structure from the group of diffeomorphisms. The formulation relies on principles of continuity of medical images as classical functions, generalizing optical flow and advection of material to diffeomorphic flow of material, the material represented by the contrast seen in the medical imaging modality such as bold MRI contrast for gray matter or fiber orientation for diffusion tensor imaging. Unifying this representation to images built at the particle and molecular biological scale has required us to move away from classical functions, to the more modern 20th century theory of nonclassical generalized functions. Mathematical measures are the proper representation as they generally reflect the property that probes from molecular biology associated to disjoint sets are additive, the basic starting point of measure theory. Changing the model from a focus on groups acting on functions to groups acting on measures allows for a unified representation that has both a metric structure at the finest scales and a unification with the tissue imaging scales.

The brain measure formulation carries with it implicitly the notion of scale space, i.e., the existence of a sequence of pairs across scales, the measure representation of the brain, and the associated scale space reproducing kernel Hilbert space of functions which correspond to the probing measurement technologies. As such, part of the prescription of the theory is a method for crossing scales and carrying information from one scale to the other. Important to this approach is that at every scale we generate a new measure; therefore, the recipe of introducing “measure norms” built from RKHS’s for measuring brain disparity is universal across the hierarchy allowing us to work simultaneously with common data structures and a common formalism. Interestingly, the measure norms do not require identical particle numbers across brains in brain space at the molecular scales.

The key modeling element of brain function is that the conditional feature probability is manipulated from the quantized features to the stochastic laws. These are the analogues of the Boltzmann distributions generalized to the complex feature spaces representing function. As they correspond to arbitrary feature spaces not necessarily Newtonian particles, we represent them simply as empirical distributions on the feature space, with the empirical measure constructed from the collapse of the fine scale to the resampled coarse scale. To model rescaling through scale space explicitly, the two kernel transformations are used allowing us to retrieve the empirical averages represented by the determinism of the stochastic law consistent with our views of the macro tissue scales. This solves the dilemma that for the quantized atomic and microscales, cell occurrence will never repeat; i.e., there is zero probability of finding a particular cell at a particular location and conditioned on finding it once it will never be found again in the exact same location in another preparation. The properties that are stable are the probability laws with associated statistics that may transfer across organisms and species.

Importantly, our introduction of the |dφ(x)| term in the action enables the crucial property that when a tissue is extended to a larger area, the total number of its basic constituents should increase accordingly and not be conserved. This is not a traditional measure transport which is mass preserving which is not a desirable feature for biological samples. Rather, we have defined a new action on measures that is reminiscent of the action on *d*-dimensonal varifolds [37, 38]. We call this property “copy and paste,” the notion being that the brain is built on basic structuring elements in their design that are conserved.

We believe that many different diffeomorphism methods can be used at multiscale. The proper coupling of the vector fields would have to be derived to determine how the different scales mix as we have done for the multiscale LDDMM formulation here based on the total kinetic energy. Also, successive refinement for the small deformation setting has been introduced in many areas associated to multigrid and basis expansions. The notion of building multiscale representation in the large deformation LDDMM setting was originally explored by Risser et al. [39] in which the kernels are represented as a sum of kernels and Sommer et al. [40] in which the kernel is represented as vector bundles. In their multiscale setting, there is a postoptimization decomposition in which the contribution of the velocity field into its different components can then each be integrated. In that multiscale setting, the basic Euler-Lagrange equation termed EPDIFF remains that of LDDMM [41]. In the setting proposed here, we separate the scales before optimization via the hierarchy of layered diffeomorphisms and use a multiscale representation of the brain hierarchy itself which is directly associated to the diffeomorphism at that scale. This gives the fundamental setting of the product group of diffeomorphisms with the Euler-Lagrange equation corresponding to the sequence of layered diffeomorphisms for multiscale LDDMM [25].

In terms of the efficiency of the multiscale representation, Figure 6 shows clearly the power of the multiscale diffeomorphism transferring microscopic and macroscopic scale properties of the brain as well as the power of crossing of scales transferring information consistently from particles to the continuum. What is striking in Figure 6 is that the deformations of the particles at the micron scale result in consistent motions of the cortical surface as a smooth global manifold. Also, the functional feature being transferred consistently from the particles via the composition of transformations $T_{1}$ and $T_{2}$ of equation (8) shows the clear pattern of the functional tau particle size being layered, a property that is hardly noticed at the molecular scale, but is clearly associated to the tissue scale.

The aggregation across scales from particle to tissue scales on lattices provides the essential link to inference on graphs. It is natural for the aggregated features on lattices with associated conditional probability laws to become the nodes in Markov random field modeling for spatial inference (see examples in spatial transcriptomics and tissue segmentation [42]). Building neighborhood relations as conditional probabilities between lattice sites from which global probability laws are constructed with the Hammersley-Clifford theorem links us to Grenander’s metric pattern theory formalisms with the atoms and conditional laws ${\left(x_{i},\mu_{x_{i}}\right)}_{i\in I}$ at any scale playing the roles of the generators.

## 4. Materials and Methods

### 4.1. Experimental and Technical Design.

The objective of this research is to unify the molecular representations of spatial transcriptomics and cellular scale histology with the tissue scales of computational anatomy for brain mapping. To accomplish this, we designed a mathematical framework for representing data at multiple scales using geometric measures as generalized functions and mapping data using geodesic flows of multiscale diffeomorphisms. We illustrate the method using several examples from human MRI and digital pathology, as well as mouse spatial transcriptomics.

### 4.2. Gaussian Kernel Varifold Norm.

Our varifold norm construction models the brain measures as elements of a Hilbert space $W^{*}$ which is dual to an RKHS *W* with a kernel $K_{W}$. Using the dual bracket notation for h∈W, $\mu \in W^{*}$ for μ a measure, then $\left(\mu |h):=\int_{{\mathbb{R}}^{d}\times ℱ}\mu (dx,df)h(x,f\right)$, and the norm becomes the integration against the kernel equation (15b) written as $∥\mu {∥}_{W^{*}}^{2}:=\left(\mu |K_{W}[\mu ]\right)$; the multiscale norm is given by $∥\mu {∥}_{W^{*}}^{2}:=\sum_{\ell \geq 0}{\left\|\mu^{\ell }\right\|}_{W_{\ell }^{*}}^{2}$.

To ensure the brain measures are elements of $W^{*}$ dual to the RKHS, the kernel $K_{W}$ is chosen to densely and continuously embed *W* in bounded continuous functions ${𝓒}_{b}\left({\mathbb{R}}^{d}\times ℱ,\mathbb{R}\right)$ so that the signed measure spaces ${ℳ}_{s}\left({\mathbb{R}}^{d}\times ℱ\right)$ are continuously embedded in $W^{*}$. An example kernel is the Gaussian kernel which satisfies this condition, the kernel taken as non-normalized, separable Gaussians with |⋅| Euclidean distance:

(29) $$k_{W}((x,f),(y,g)):=\exp\left(-\frac{1}{2\sigma_{x}^{2}}|x-y{|}^{2}\right)\exp\left(-\frac{1}{2\sigma_{f}^{2}}|f-g{|}^{2}\right).$$

Measures $\mu^{a}=\sum_{i\in I}w_{i}^{a}\delta_{x_{i}^{a}}⊗\delta_{f_{i}^{a}}$, $\mu^{b}=\sum_{j\in J}w^{b}\delta_{x_{j}^{b}}⊗\delta_{f_{i}^{b}}$ have norm-square

$${\left\|\mu^{a}-\mu^{b}\right\|}_{W^{*}}^{2}$$

(30) $$=\sum_{i,i^{\prime }\in I}w_{i}^{a}w_{i^{\prime }}^{a}\exp\left(-\frac{1}{2\sigma_{x}^{2}}{\left|x_{i}^{a}-x_{i^{\prime }}^{a}\right|}^{2}\right)\exp\left(-\frac{1}{2\sigma_{f}^{2}}{\left|f_{i}^{a}-f_{i^{\prime }}^{a}\right|}^{2}\right)\;-2\sum_{i\in I,j\in J}w_{i}^{a}w_{j}^{b}\exp\left(-\frac{1}{2\sigma_{x}^{2}}{\left|x_{i}^{a}-x_{j}^{b}\right|}^{2}\right)\exp\left(-\frac{1}{2\sigma_{f}^{2}}{\left|f_{i}^{a}-f_{j}^{b}\right|}^{2}\right)\;+\sum_{j,j^{\prime }\in J}w_{j}^{b}w_{j^{\prime }}^{b}\exp\left(-\frac{1}{2\sigma_{x}^{2}}{\left|x_{j}^{b}-x_{j^{\prime }}^{b}\right|}^{2}\right)\exp\left(-\frac{1}{2\sigma_{f}^{2}}{\left|f_{j}^{b}-f_{j^{\prime }}^{b}\right|}^{2}\right).$$

For data with position information but no features (tau tangle locations), each $f_{i},f_{j}$ is constant with exponential terms all 1.

### 4.3. The Riemannian Distance Metric on the Multiscale Group.

The diffeomorphism group acts on the hierarchy φ⋅μ component-wise equation (11c) with the multiscale group product

(31) $$G_{k}=\underset{\ell_{\text{max}}\text{times}}{\underset{︸}{G_{k}\times \cdots \times G_{k}}},$$

with elements $\varphi \in G_{k}$ satisfying the law of composition component-wise $\varphi \circ \varphi^{\prime }={\left(\varphi^{\ell }\circ \varphi^{\prime \ell }\right)}_{\ell \geq 0}$. The group $G_{k}$ supporting k-derivatives of the diffeomorphisms builds from ${𝓒}_{0}^{k}\left({\mathbb{R}}^{d},{\mathbb{R}}^{d}\right)$ a space of k-times continuously differentiable vector fields vanishing at infinity and its partial derivatives of order p≤k intersecting with diffeomorphisms with 1-derivative:

(32) $$G_{k}=\left(\text{id}+{𝓒}_{0}^{k}\left({\mathbb{R}}^{d},{\mathbb{R}}^{d}\right)\right)\cap \;{\text{Diff}}^{1}\left({\mathbb{R}}^{d},{\mathbb{R}}^{d}\right).$$

Dynamics occurs via group action generated as a dynamical system in which the multiscale control $t\mapsto u_{t}:={\left(u_{t}^{\ell }\right)}_{\ell \geq 0}$ follows $t\mapsto \varphi_{t}$ satisfying ${\dot{\varphi }}_{t}=u_{t}\circ \varphi_{t}$ of (12a). The control of (12a). The control is in the product $V={∐}_{\ell \geq 0}V_{\ell }$ each space an RKHS with norm-square $∥\cdot {∥}_{V}^{2}=\sum_{\ell \geq 0}∥\cdot {∥}_{V_{\ell }}^{2}$ selected to control the smoothness of the vector fields. The hierarchy of spaces is organized as a sequence of continuous embeddings, $V_{0}↪\cdots ↪V_{\ell_{\max}}$, where $V_{\ell_{\max}}$ is an additional layer containing the others with $V_{\ell_{\max}}={𝓒}_{0}^{m}\left({\mathbb{R}}^{d},{\mathbb{R}}^{d}\right)$ defined as a space of *m*-times continuously differentiable vector fields vanishing at infinity as well all its partial derivatives of order p≤m.

The hierarchy is connected via successive refinements $u^{\ell }=u^{\ell -1}+v^{\ell },u^{0}=v^{0}$ expressed via the continuous linear operator A:V→V with v=Au. The control process ${\left(u_{t}\right)}_{0\leq t\leq 1}\in L^{2}([0,1],V)$ has finite square integral with total energy

(33) $${ℰ}_{A}\left({\left(u_{t}\right)}_{0\leq t\leq 1}\right)=\frac{1}{2}\int_{0}^{1}{\left\|Au_{t}\right\|}_{V}^{2}dt.$$

Optimal curves which minimize the integrated energy ${ℰ}_{A}$ between any two fixed boundary conditions (BC) $\varphi_{0}=\text{Id}$ and $\varphi_{1}$ which is accessible with a path of finite energy extend the LDDMM setting [26] to a hierarchy of diffeomorphisms and describe a geodesic for an associated Riemannian metric and multiscale LDDMM [25] on $G_{k}$:

(34) $$d_{G_{k}}{\left(\mathrm{Id},\varphi_{1}\right)}^{2}:=\underset{\begin{array}{c} \begin{array}{c} {\left(u_{t^{\prime }}\right)}_{0\leq t\leq 1}\in L^{2}([0,1],V):\\ {\dot{\varphi }}_{t}=u_{t^{\circ }}\varphi_{t},\;\text{with BC}\;\varphi_{0}=\mathrm{Id},\varphi_{1} \end{array} \end{array}}{\min}\int_{0}^{1}{\left\|Au_{t}\right\|}_{V}^{2}dt.$$

Existence of solutions for minimizers over ${\left(u_{t}\right)}_{0\leq t\leq 1}$ of (34) when $d_{G_{k}}\left(\mathrm{Id},\varphi_{1}\right)$ is finite can be established when m≥k≥1.

### 4.4. Geodesic Multiscale LDDMM via Hamiltonian Control.

The shape of Brainspace is given by its geodesics. We use Hamiltonian control to generate the geodesics.

The Hamiltonian method reduces the parameterization of the vector field to the dynamics of the particles that encode the flow of states (17a). We write the dynamics explicitly as a linear function of the control, and define the flow of the measures indexed by the dynamical state:

(35a) $$\begin{array}{l} {\dot{q}}_{t}=\xi_{q_{t}}\left(u_{t}\right):={\left(\xi_{q_{t}^{\ell }}\left(u_{t}^{\ell }\right)\right)}_{\ell \geq 0},\\ \;\text{with}\;{\dot{q}}_{t}=\{\begin{array}{l} {\left(u_{t}\left(x_{i,t}\right),w_{i,t}\text{div}\;u_{t}\left(x_{i,t}\right)\right)}_{i\in I},\\ {\left(u_{t}\left(\varphi_{t}(x)\right),w_{t}(x)\text{div}\;u_{t}\left(\varphi_{t}(x)\right)\right)}_{x\in {\mathbb{R}}^{d}}, \end{array} \end{array}$$

(35b) $$\begin{array}{l} \mu \left(q_{t}\right):={\left(\mu \left(q_{t}^{\ell }\right)\right)}_{\ell \geq 0},\\ \;\text{with}\;\mu \left(q_{t}\right):=\{\begin{array}{l} \sum_{i\in I}w_{i,t}\delta_{x_{i,t}}⊗\delta_{f_{i}},\\ \int_{{\mathbb{R}}^{d}}\rho (x)w_{t}(x)\delta_{\varphi_{t}(x)}⊗\delta_{f(x)}dx. \end{array} \end{array}$$

The control problem satisfying (16) reparameterized in the states becomes, for α>0,

(36) $$\underset{\begin{array}{l} {\left(u_{t}\right)}_{0\leq t\leq 1}\in L^{2}([0,1],V)\\ {\dot{q}}_{t}=\xi_{q_{t}}\left(u_{t}\right),\mu \left(q_{0}\right)=\mu \end{array}}{\min}\frac{1}{2}\int_{0}^{1}{\left\|Au_{t}\right\|}_{V}^{2}dt+\frac{\alpha }{2}{\|\mu \left(q_{1}\right)-\mu_{\text{obs}}\|}_{\text{W}*}^{2}.$$

Hamiltonian control for particles and tissues introduces the costates $t\mapsto p_{t}$ via the Hamiltonian

(37a) $$ℋ(q,p,u):=\left(p|\xi_{q}(u)\right)-\frac{1}{2}∥Au{∥}_{V}^{2},$$

(37b) $$\text{with}\;\left(p|\xi_{q}(u)\right)=\{\begin{array}{l} \sum_{\ell \geq 0}\sum_{i\in I_{\ell }}\left({\langle p_{i}^{x,\ell },u^{\ell }\left(x_{i}^{\ell }\right)\rangle }_{{\mathbb{R}}^{d}}+p_{i}^{w,\ell }w_{i}^{\ell }\text{div}\;u^{\ell }\left(x_{i}^{\ell }\right)\right),\\ \sum_{\ell \geq 0}\int_{{\mathbb{R}}^{d}}({\langle p^{\varphi ,\ell }(x),u^{\ell }\left(\varphi^{\ell }(x)\right)\rangle }_{{\mathbb{R}}^{d}}\\ \;+p^{w,\ell }(x)w^{\ell }(x)\text{div}\;u^{\ell }\left(\varphi^{\ell }(x)\right))dx. \end{array}$$

Under the assumption $V_{\ell_{\max}}\to {𝓒}_{0}^{k+2}\left({\mathbb{R}}^{d},{\mathbb{R}}^{d}\right)$ the Pontryagin maximum [22] with k≥1 gives the optimal control for all scales ℓ satisfying

(38) $$\{\begin{array}{l} \partial_{u^{\ell }}ℋ(q,p,u)=0,\\ {\dot{q}}^{\ell }=\partial_{p^{\ell }}ℋ(q,p,u),\\ {\dot{p}}^{\ell }=-\partial_{q^{\ell }}ℋ(q,p,u). \end{array}$$

#### Statement 1.

Geodesics of particles. Assume that $V_{\ell_{\max}}={𝓒}_{0}^{m}\left({\mathbb{R}}^{d},{\mathbb{R}}^{d}\right)$ with m≥k+2 and k≥1. If ${\left(u_{t}\right)}_{0\leq t\leq 1}$ is a solution of the optimal control problem (36), then there exists a time-dependent costate $t\mapsto p_{t}={\left(p_{i,t}^{\ell }\right)}_{i\in I_{\ell },\ell \geq 0}$ for all ℓ satisfying

(39a) $$\{\begin{array}{l} {\dot{q}}_{i,t}^{\ell }=\left(u_{t}^{\ell }\left(x_{i,t}^{\ell }\right),w_{i,t}^{\ell }\;\text{div}\;u_{t}^{\ell }\left(x_{i,t}^{\ell }\right)\right),\\ {\dot{p}}_{i,t}^{x,\ell }=-{\left(du_{t}^{\ell }\right)}^{T}\left(x_{i,t}^{\ell }\right)p_{i,t}^{x,\ell }-p_{i,t}^{w,\ell }w_{i,t}^{\ell }\nabla \left(\text{div}\;u_{t}^{\ell }\right)\left(x_{i,t}^{\ell }\right),\\ {\dot{p}}_{i,t}^{w,\ell }=-p_{i,t}^{w,\ell }\;\text{div}\;u_{t}^{\ell }\left(x_{i,t}^{\ell }\right). \end{array}$$

The optimal control satisfying $\partial_{u^{\ell }}ℋ(q,p,u)=0$ for any ℓ≥0 and $v=Au\left(v^{\ell_{\max}}=0\right)$ is given by (18a).

#### Geodesics of Tissue.

Assume that $V_{\ell_{\max}}={𝓒}_{0}^{m}\left({\mathbb{R}}^{d},{\mathbb{R}}^{d}\right)$ with m≥k+2,k≥1. If ${\left(u_{t}\right)}_{0\leq t\leq 1}$ solves the optimal control problem (36) then the time-dependent costate $t\mapsto p_{t}={\left(p_{t}^{\ell }(x)\right)}_{x\in {\mathbb{R}}^{d},\ell \geq 0}$ for all ℓ satisfies

(39b) $$\{\begin{array}{l} {\dot{q}}_{t}^{\ell }(x)=\left(u_{t}^{\ell }\left(\varphi_{t}^{\ell }(x)\right),w_{t}^{\ell }(x)\;\text{div}\;u_{t}^{\ell }\left(\varphi_{t}^{\ell }(x)\right)\right),\\ {\dot{p}}_{t}^{\varphi ,\ell }(x)=-{\left(du_{t}^{\ell }\right)}^{T}\left(\varphi_{t}^{\ell }(x)\right)p_{t}^{\varphi ,\ell }(x)\\ \;-p_{t}^{w,\ell }(x)w_{t}^{\ell }(x)\nabla \left(\text{div}\;u_{t}^{\ell }\right)\left(\varphi_{t}^{\ell }(x)\right),\\ {\dot{p}}_{t}^{w,\ell }(x)=-p_{t}^{w,\ell }(x)\text{div}\;u_{t}^{\ell }\left(\varphi_{t}^{\ell }(x)\right). \end{array}$$

The optimal controls satisfying $\partial_{u^{\ell }}ℋ(q,p,u)=0$ for any ℓ≥0 and $v=Au\left(v^{\ell_{\max}}=0\right)$ are given by (19a).

See Appendix A for proof of differential equations.

#### Statement 2.

(Integral equations for Hamiltonian Momentum of particles). Assuming $q\mapsto U(q):=(\alpha /2){\left\|\mu (q)-\mu_{\text{obs}}\right\|}_{W^{*}}^{2}$ is ${𝓒}^{1}$ in q, the geodesic costate for particles flowing from t=0,1 satisfies

(40) $$\{\begin{array}{l} p_{i,t}^{x,\ell }={\left(d\varphi_{t,0}^{\ell }\right)}^{T}\left(x_{i,t}^{\ell }\right)p_{i,0}^{x,\ell }-p_{i,0}^{w,\ell }w_{i,0}^{\ell }\int_{0}^{t}{\left(d\varphi_{t,s}^{\ell }\right)}^{T}\left(x_{i,t}^{\ell }\right)\nabla \left(\text{div}\;u_{s}^{\ell }\right)\left(x_{i,s}^{\ell }\right)ds,\\ p_{i,t}^{w,\ell }=p_{i,0}^{w,\ell }\left|d\varphi_{t,0}^{\ell }\right|\left(x_{i,t}^{\ell }\right),\\ p_{i,1}^{x,\ell }={\left(d\varphi_{t,s}^{\ell }\right)}^{T}\left(x_{i,t}^{\ell }\right)p_{i,1}^{x}+p_{i,1}^{w}w_{i,1}^{\ell }\int_{t}^{1}{\left(d\varphi_{t,s}\right)}^{T}\left(x_{i,t}^{\ell }\right)\nabla \left(\text{div}\;u_{s}^{\ell }\right)\left(x_{i,s}^{\ell }\right)ds,\\ p_{i,t}^{w,\ell }=p_{i,1}^{w,\ell }\left|d\varphi_{t,s}^{\ell }\right|\left(x_{i,t}^{\ell }\right). \end{array}$$

As proven in Appendix B, the particle integral equations of (40) and (18b) satisfy (39a). The second set of dense tissue integral equations (19b) satisfies (39b) by a similar argument.

### 4.5. Gradients of the Endpoint Varifold Matching Norm.

The gradients of the matching endpoints require us to compute the variation ${(d/d\epsilon )U\left(\varphi_{1}(\epsilon ),w_{1}(\epsilon )\right)|}_{\epsilon =0}$ requiring *d U* as a function of φ is ${𝓒}^{1}$ for $\varphi \in G_{k}\subset {\text{Diff}}_{\text{id}}^{k}\left({\mathbb{R}}^{d},{\mathbb{R}}^{d}\right)$; this requires k≥1.

The gradients (20b) are rewritten using the state $q_{t}={\left(x_{i,t},w_{i,t}\right)}_{i\in I}$ to define the norm-square in terms of $h_{q}$ continuously differentiable in *x* and bounded ${𝓒}_{b}^{1,0}$ determining the smooth gradients:

(41) $$h_{q_{1}}(y,z):=\alpha K_{W}\left[\mu \left(q_{1}\right)-\mu_{\text{obs}}\right](y,z),\;(y,z)\in {\mathbb{R}}^{\text{d}}\times ℱ\text{.}$$

We take the variation ${\left(d/d\epsilon )U(q(\varepsilon )\right)|}_{\varepsilon =0}$ varying each term $q_{1}(\varepsilon )={\left(x_{i,1}+\epsilon \psi_{i}^{x},w_{i,1}+\epsilon \psi_{i}^{w}\right)}_{i\in I}$ with dependence on ℓ-scale implied:

(42) $${\frac{d}{d\epsilon }\frac{\alpha }{2}{\left\|\mu \left(q_{1}(\epsilon )\right)-\mu_{\text{obs}}\right\|}_{W^{*}}^{2}|}_{\epsilon =0}.\;\;={\frac{d}{d\epsilon }\frac{\alpha }{2}\left(\mu \left(q_{1}(\epsilon )\right)-\mu_{\text{obs}}|K_{W}\left[\mu \left(q_{1}(\epsilon )\right)-\mu_{\text{obs}}\right]\right)|}_{\epsilon =0}\;\;={\frac{d}{d\epsilon }\left(\mu \left(q_{1}(\epsilon )\right)|\alpha K_{W}\left[\mu \left(q_{1}\right)-\mu_{\text{obs}}\right]\right)|}_{\epsilon =0}\;\;={\sum_{i\in I}\frac{d}{d\epsilon }\left(\left(w_{i,1}+\epsilon \psi_{i}^{w}\right)h_{q_{1}}\left(x_{i,1}+\epsilon \psi_{i}^{x},f_{i}\right)\right)|}_{\epsilon =0}\;\;=\sum_{i\in I}\left({\left\|w_{i,1}\nabla_{x_{i}}h_{q_{1}}\left(x_{i,1},f_{i}\right),\psi_{i}^{x}\right\|}_{R^{d}}+h_{q_{1}}\left(x_{i,1},f_{i}\right)\psi_{i}^{w}\right))=0.$$

The gradients (20c) for tissue have $\mu =\int_{{\mathbb{R}}^{d}}w(x)\rho (x)\delta_{x}⊗\mu_{x}dx$ and $\mu \left(q_{t}\right)=\int_{{\mathbb{R}}^{d}}w_{t}(x)\rho (x)\delta_{\varphi_{t}(x)}⊗\mu_{x}dx$ with $q_{t}:=\left(\varphi_{t},w_{t}=w\left|d\varphi_{t}\right|\right)$. The average of $h_{q_{1}}$ over the feature space determines the boundary term variation.

With $q_{1}(\epsilon )=\left(\varphi_{1}(\epsilon ),w_{1}(\epsilon )\right)$, take the variation $\varphi_{1}(\epsilon )=\varphi_{1}+\epsilon \psi^{\varphi }$ and $w_{1}(\epsilon )=w_{1}+\epsilon \psi^{w}$:

(43) $${\frac{d}{d\epsilon }\frac{\alpha }{2}{\left\|\mu \left(q_{1}(\epsilon )\right)-\mu_{\text{obs}}\right\|}_{W^{*}}^{2}|}_{\epsilon =0}\;\;={\frac{d}{d\epsilon }\frac{\alpha }{2}\left(\mu \left(q_{1}(\epsilon )\right)-\mu_{\text{obs}}|K_{W}\left[\mu \left(q_{1}(\epsilon )\right)-\mu_{\text{obs}}\right]\right)|}_{\epsilon =0}\;\;={\frac{d}{d\epsilon }\left(\mu \left(q_{1}(\epsilon )\right)|\alpha K_{W}\left[\mu \left(q_{1}\right)-\mu_{\text{obs}}\right]\right)|}_{\epsilon =0}\;\;={\frac{d}{d\epsilon }\left(\mu \left(\varphi_{1}(\epsilon ),w_{1}(\epsilon )\right)|h_{q_{1}}\right)|}_{\epsilon =0}\;\;=\frac{d}{d\epsilon }(\int_{{\mathbb{R}}^{d}\times ℱ}h_{q_{1}}(y,f)\mu \left(\varphi_{1}(\epsilon ),w_{1}\right)(dy,df)\;\;+\int_{{\mathbb{R}}^{d}\times ℱ}h_{q_{1}}(y,f)\mu \left(\varphi_{1},w_{1}(\epsilon )\right)(dy,df)){|}_{\epsilon =0}\;\;=\frac{d}{d\epsilon }\int_{{\mathbb{R}}^{d}\times ℱ}h_{q_{1}}(y,f)(\int_{{\mathbb{R}}^{d}}w_{1}(x)\rho (x)\delta_{\varphi_{1}(\epsilon )(x)}(dy)\mu_{x}(df)dx\;\;+\int_{{\mathbb{R}}^{d}}w_{1}(\epsilon ,x)\rho (x)\delta_{\varphi_{1}(x)}(dy)\mu_{x}(df)dx){|}_{\epsilon =0}\;\;={\int_{{\mathbb{R}}^{d}\times ℱ}w_{1}(x)\rho (x)\frac{d}{d\epsilon }h_{q_{1}}\left(\varphi_{1}(\epsilon )(x),f\right)|}_{\epsilon =0}\mu_{x}(df)dx\;\;+{\int_{{\mathbb{R}}^{d}\times ℱ}\frac{d}{d\epsilon }w_{1}(\epsilon ,x)|}_{\epsilon =0}\rho (x)h_{q_{1}}\left(\varphi_{1}(x),f\right)\mu_{x}(df)dx\;\;=\int_{{\mathbb{R}}^{d}}\langle w_{1}(x)\rho (x)\left(\int_{ℱ}\nabla_{\varphi }h_{q_{1}}\left(\varphi_{1}(x),f\right)\mu_{x}(df)\right),\psi^{\varphi }{(x)\rangle }_{{\mathbb{R}}^{d}}dx\;\;+\int_{{\mathbb{R}}^{d}}\rho (x)\left(\int_{ℱ}h_{q_{1}}\left(\varphi_{1}(x),f\right)\mu_{x}(df)\right)\psi^{w}(x)dx=0.$$

## Data Availability

The major contribution of this work is a mathematical and computational framework for unifying the molecular and tissue scales appropriate for mapping and understanding modern neuroimaging data. Specific datasets shown in examples are for illustrative purposes but can be made available upon request.

## Appendix

### A. Hamiltonian Control Statement

We take for our RKHS $V_{\ell },\ell \geq 0$ with the operators $L_{\ell }:V_{\ell }\to V_{\ell }^{*}$ defining the isometries such that the inner products ${\langle \cdot ,\cdot \rangle }_{V_{\ell }}$ satisfy for any $u^{\prime \ell },v^{\prime \ell }\in V_{\ell }$:

(A.1)
$${\langle u^{\prime \ell },v^{\prime \ell }\rangle }_{V_{\ell }}=\left(L_{\ell }u^{\prime \ell }|v^{\prime \ell }\right).$$

#### Statement 1.

We assume that $V_{\ell_{\max}}={𝓒}_{0}^{m}\left({\mathbb{R}}^{d},{\mathbb{R}}^{d}\right)$ with m≥k+2 and k≥1.

If ${\left(u_{t}\right)}_{0\leq t\leq 1}$ is a solution of the optimal control problem (36), then for particles there exists time-dependent costate $t\mapsto p_{t}={\left(p_{i,t}^{\ell }\right)}_{i\in I_{\ell },\ell \geq 0}$ such that all ℓ satisfies

(A.2a)
$$\{\begin{array}{l} {\dot{q}}_{i,t}^{\ell }=\left(u_{t}^{\ell }\left(x_{i,t}^{\ell }\right),w_{i,t}^{\ell }\text{div}\;u_{t}^{\ell }\left(x_{i,t}^{\ell }\right)\right),\\ {\dot{p}}_{i,t}^{x,\ell }=-{\left(du_{t}^{\ell }\right)}^{T}\left(x_{i,t}^{\ell }\right)p_{i,t}^{x,\ell }-p_{i,t}^{w,\ell }w_{i,t}^{\ell }\nabla \left(\text{div}\;u_{t}^{\ell }\right)\left(x_{i,t}^{\ell }\right),\\ {\dot{p}}_{i,t}^{w,\ell }=-p_{i,t}^{w,\ell }\text{div}\;u_{t}^{\ell }\left(x_{i,t}^{\ell }\right). \end{array}$$

The optimal control for particles satisfies for every scale $\partial_{u^{\ell }}ℋ(q,p,u)=0$ with $v=Au\left(v^{\ell_{\max}}=0\right)$:

(A.2b)
$$\{\begin{array}{l} v_{t}^{\ell }(\cdot )=u_{t}^{\ell }(\cdot )-u_{t}^{\ell -1}(\cdot )=\sum_{j\geq \ell }\sum_{i\in I_{j}}\left(g_{\ell }\left(x_{i,t}^{j},\cdot \right)p_{i,t}^{x,j}+p_{i,t}^{w,j}\nabla_{1}g_{\ell }\left(x_{i,t}^{j},\cdot \right)\right),\\ u_{t}^{\ell }(\cdot )=\sum_{j\geq 0}\sum_{i\in I_{j}}\left({\bar{g}}_{\ell \wedge j}\left(x_{i,t}^{j}\cdot \right)p_{i,t}^{x,j}+p_{i,t}^{w,j}w_{i,t}^{j}\nabla_{1}{\bar{g}}_{\ell \wedge j}\left(x_{i,t}^{j},\cdot \right)\right), \end{array}$$

with ${\bar{g}}_{\ell }:=\sum_{j=0}^{\ell }g_{j}$ and $\nabla_{1}g_{\ell }(a,b)$ denoting the gradient of $g_{\ell }$ with respect to the first variable *a* and with shorthand notation ≥ℓ to mean upper bounded by $\ell_{\max}$.

#### Statement 2.

We assume that $V_{\ell_{\max}}={𝓒}_{0}^{m}\left({\mathbb{R}}^{d},{\mathbb{R}}^{d}\right)$ with m≥k+2 and k≥1.

If ${\left(u_{t}\right)}_{0\leq t\leq 1}$ is a solution of the optimal control problem (36), then for tissue there exists time-dependent costate $\left(t\mapsto p_{t}={\left(p_{t}^{\ell }(x)\right)}_{x\in {\mathbb{R}}^{d},\ell \geq 0}\right)$ such that all ℓ satisfies

(A.3a)
$$\{\begin{array}{l} {\dot{q}}_{t}^{\ell }(x)=\left(u_{t}^{\ell }\left(\varphi_{t}^{\ell }(x)\right),w_{t}^{\ell }(x)\text{div}\;u_{t}^{\ell }\left(\varphi_{t}^{\ell }(x)\right)\right),\\ {\dot{p}}_{t}^{\varphi ,\ell }(x)=-{\left(du_{t}^{\ell }\right)}^{T}\left(\varphi_{t}^{\ell }(x)\right)p_{t}^{\varphi ,\ell }(x)-p_{t}^{w,\ell }(x)w_{t}^{\ell }(x)\nabla \left(\text{div}\;u_{t}^{\ell }\right)\left(\varphi_{t}^{\ell }(x)\right),\\ {\dot{p}}_{t}^{w,\ell }(x)=-p_{t}^{w,\ell }(x)\;\text{div}\;u_{t}^{\ell }\left(\varphi_{t}^{\ell }(x)\right). \end{array}$$

The optimal control for tissue satisfies for every scale $\partial_{u^{\ell }}ℋ(q,p,u)=0$ with

(A.3b)
$$\{\begin{array}{l} v_{t}^{\ell }(\cdot )=u_{t}^{\ell }(\cdot )-u_{t}^{\ell -1}(\cdot )\\ \;=\sum_{j\geq \ell }\int_{{\mathbb{R}}^{d}}\left(g_{\ell }\left(\varphi_{t}^{j}(y),\cdot \right)p_{t}^{\varphi ,j}(y)+p_{t}^{w,j}(y)w_{t}^{j}(y)\nabla_{1}g_{\ell }\left(\varphi_{t}^{j}(y),\cdot \right)\right)dy,\\ u_{t}^{\ell }(\cdot )=\sum_{j\geq 0}\int_{{\mathbb{R}}^{d}}\left({\bar{g}}_{\ell \wedge j}\left(\varphi_{t}^{j}(y),\cdot \right)p_{t}^{\varphi ,j}(y)+p_{t}^{w,j}(y)w_{t}^{j}(y)\nabla_{1}{\bar{g}}_{\ell \wedge j}\left(\varphi_{t}^{j}(y),\cdot \right)\right)dy. \end{array}$$

*Proof.* Under the assumption $V_{\ell_{\max}}\to {𝓒}_{0}^{k+2}\left({\mathbb{R}}^{d},{\mathbb{R}}^{d}\right)$, then we have $\left(u,q)\mapsto \xi_{q}(u\right)$ is ${𝓒}^{2}$ and standard results of optimal control theory applying the Pontryagin maximum principle [22] gives

(A.4)
$$\{\begin{array}{l} \dot{q}=\partial_{p}ℋ,\\ \dot{p}=-\partial_{q}ℋ,\\ \partial_{u}ℋ=0. \end{array}$$

Showing Statement 1, the state and Hamiltonian momentum variations are at the same scale for all ℓ≥0; therefore, we omit superscripts ℓ. For particles, the variations at any scale ℓ, $\partial_{p^{\ell }}ℋ$, and $\partial_{q^{\ell }}ℋ$ vary $p_{i}^{x}(\epsilon )=p_{i}^{x}+\epsilon \psi_{i}^{p_{x}}$, $p_{i}^{w}(\epsilon )=p_{i}^{w}+\epsilon \psi_{i}^{p_{w}}$, and $x_{i}(\epsilon )=x_{i}+\epsilon \psi_{i}^{x}$, $w_{i}(\epsilon )=w_{i}+\epsilon \psi_{i}^{w}$, respectively, giving the state velocities and momentum differential equations of (A.2a):

$$\begin{array}{l} \frac{d}{d\epsilon }{\left(p(\epsilon )|\xi_{q}(u)\right)|}_{\epsilon =0}\\ \;={\frac{d}{d\epsilon }\sum_{i\in I}\left({\langle p_{i}^{x}+\epsilon \psi_{i}^{p_{x}},u\left(x_{i}\right)\rangle }_{{\mathbb{R}}^{d}}+\left(p_{i}^{w}+\epsilon \psi_{i}^{p_{w}}\right)w_{i}\;\text{div}\;u\left(x_{i}\right)\right)|}_{\epsilon =0}\\ \;=\sum_{i\in I}\left({\langle \psi_{i}^{p_{x}},u\left(x_{i}\right)\rangle }_{{\mathbb{R}}^{d}}+\psi_{i}^{p_{w}}w_{i}\;\text{div}\;u\left(x_{i}\right)\right), \end{array}$$

(A.5)
$$\begin{array}{l} \frac{d}{d\epsilon }{\left(p|\xi_{q(\epsilon )}(u)\right)|}_{\epsilon =0}\\ ={\frac{d}{d\epsilon }\sum_{i\in I}\left({\langle p_{i}^{x},u\left(x_{i}(\epsilon )\right)\rangle }_{{\mathbb{R}}^{d}}+p_{i}^{w}w_{i}(\epsilon )\;\text{div}\;u\left(x_{i}(\epsilon )\right)\right)|}_{\epsilon =0}\\ =\sum_{i\in I}({\langle p_{i}^{x},du\left(x_{i}\right)\psi_{i}^{x}\rangle }_{{\mathbb{R}}^{d}}+p_{i}^{w}w_{i}{\langle \nabla \;\text{div}\;u\left(x_{i}\right),\psi_{i}^{x}\rangle }_{{\mathbb{R}}^{d}}\\ \;+p_{i}^{w}\;\text{div}\;u\left(x_{i}\right)\psi_{i}^{w}). \end{array}$$

The tissue proof (A.3a) is a similar argument. We prove the continuum tissue case of (A.3b) for the optimal control since it is the multiscale version of the original LDDMM conservation of momentum equations derived in [25, 41]. The particle proof for (A.2b) is similar. Calculating $\partial_{u^{\ell }}ℋ=0$, define $u^{\ell }(\epsilon )=u^{\ell }+\epsilon \psi^{u}$ for $\psi^{u}\in V_{\ell_{\max}}$, implying $v^{\ell +1}(\epsilon )=v^{\ell +1}-\epsilon \psi^{u}$, $v^{\ell }(\epsilon )=v^{\ell }+\epsilon \psi^{u}$:

(A.6)
$${\frac{d}{d\epsilon }\left(\left(p^{\ell }|\xi_{q^{\ell }}\left(u^{\ell }(\epsilon )\right)\right)-\frac{1}{2}\sum_{j=\ell }^{\ell +1}\left(L_{j}v^{j}(\epsilon )|v^{j}(\epsilon )\right)\right)|}_{\epsilon =0}\;\;=\frac{d}{d\epsilon }(\int \left({\langle p^{\varphi ,\ell },u^{\ell }(\epsilon )\circ \varphi^{\ell }\rangle }_{{\mathbb{R}}^{d}}+p^{w,\ell }w^{\ell }\text{div}\;u^{\ell }(\epsilon )\circ \varphi^{\ell }\right)dx\;\;-\frac{1}{2}\sum_{j=\ell }^{\ell +1}\left(L_{j}v^{j}(\epsilon )|v^{j}(\epsilon )\right)){|}_{\epsilon =0}\;\;=\int_{{\mathbb{R}}^{d}}\left({\langle p^{\varphi ,\ell },\psi^{u}\circ \varphi^{\ell }\rangle }_{{\mathbb{R}}^{d}}+p^{w,\ell }w^{\ell }\text{div}\;\psi^{u}\circ \varphi^{\ell }\right)dx\;\;-\left(L_{\ell }v^{\ell }-L_{\ell +1}v^{\ell +1}|\psi^{u}\right)=0.$$

The Eulerian momentum for the system at each scale is $L_{\ell }v^{\ell }$ and is conserved as proven in [25]. Summing the momentum difference $L_{j}v^{j}-L_{j+1}v^{j+1}$ from j≥ℓ gives

(A.7)
$$\left(L_{\ell }v^{\ell }|\psi^{u}\right)=\sum_{j\geq \ell }\int_{{\mathbb{R}}^{d}}\left({\langle p^{\varphi ,j},\psi^{u}\circ \varphi^{j}\rangle }_{R^{d}}+p^{w,j}w^{j}\text{div}\;\psi^{u}\circ \varphi^{j}\right)dx.$$

We have for $\alpha \in {\mathbb{R}}^{d}$ then $\left(\alpha ⊗\delta_{x}|\psi \right)={\langle \alpha ,\psi (x)\rangle }_{{\mathbb{R}}^{d}}$ and $\left(\delta_{x}^{\text{div}\;}|\psi \right)=\text{div}\;\psi (x)$.

This gives

(A.8)
$$L_{\ell }v^{\ell }=\sum_{j=\ell }\int_{R^{d}}\left(p^{\varphi ,j}(x)⊗\delta_{\varphi^{j}(x)}+p^{w,j}(x)\delta_{\varphi^{j}(x)}^{\text{div}}\right)dx.$$

Using the definition of the Green’s kernel gives $K_{\ell }\left[\delta_{\varphi^{j}}^{\text{div}}(x)\right]=\nabla_{1}g_{\ell }\left(\varphi^{j}(x),\cdot \right)$ and $K_{\ell }\left[p^{\varphi ,j}⊗\delta_{\varphi^{j}(x)}\right]=g_{\ell }\left(\varphi^{j}(x),\cdot \right)p^{\varphi ,j}$ implies the result for $v^{\ell }$ of (A.3b). Since $u^{\ell }=\sum_{j=0}^{\ell }v^{j}$ we deduce the second line of (A.3b) for the optimal control.

### B. Hamiltonian Costate Momentum Integrable Dynamics

We omit the superscripts ℓ below since costates and states and flows are at the same scale.

#### Statement 3.

Assume $q\mapsto U(q):=(\alpha /2){\left\|\mu (q)-\mu_{\text{obs}}\right\|}_{W^{*}}^{2}$ is ${𝓒}^{1}$ in q then, the costate integral equation (18b) for particles flowing from t=1 solves the Hamiltonian differential equations $\left({\dot{p}}^{x},{\dot{p}}^{w}\right)$ of (A.2a) at every scale ℓ≥0; the integral equations flowing from t=0 satisfy

(B.1)
$$\{\begin{array}{l} p_{i,t}^{x}={\left(d\varphi_{t,0}\right)}^{T}\left(x_{i,t}\right)p_{i,0}^{x}-p_{i,0}^{w}w_{i,0}\int_{0}^{t}{\left(d\varphi_{t,s}\right)}^{T}\left(x_{i,t}\right)\nabla \left(\text{div}\;u_{s}\right)\left(x_{i,s}\right)ds,\\ p_{i,t}^{w}=p_{i,0}^{w}\left|d\varphi_{t,0}\right|\left(x_{i,t}\right). \end{array}$$

*Proof.* We show the proof for particles, with the tissue proof a similar argument. First for t=0, take $p_{i,t}^{w}=p_{i,0}^{w}\left|d\varphi_{t,0}\right|\left(x_{i,t}\right)$ of (B.1) and show that it satisfies (A.2a). For $w_{i,t}=w_{i}\left|d\varphi_{t}\right|\left(x_{i}\right)$, $x_{i,t}=\varphi_{t}\left(x_{i}\right)$ then

(B.2)
$$\frac{d}{dt}w_{i,t}p_{i,t}^{w}=\frac{d}{dt}w_{i,0}\left|d\varphi_{t}\right|\left(x_{i}\right)p_{i,0}^{w}\left|d\varphi_{t}^{-1}\right|\left(x_{i,t}\right)=0,$$

which implies ${\dot{p}}_{i,t}^{w}=-\left(p_{i,t}^{w}{\dot{w}}_{i,t}/w_{i,t}\right)=-p_{i,t}^{w}\;\text{div}\;u_{t}\left(x_{i,t}\right)$. □

Now prove $p_{i,t}^{x}$ of (B.1) satisfies (A.2a); rewrite the integral solution using $\varphi_{t,s}:=\varphi_{s}\circ \varphi_{t}^{-1}$ and the identity

(B.3)
$$\begin{array}{l} p_{i,t}^{x}={\left(d\varphi_{t}^{-1}\right)}^{T}\circ \varphi_{t}\left(x_{i}\right)p_{i,0}^{x}\\ \;-p_{i,0}^{w}w_{i,0}\int_{0}^{t}{\left(d\varphi_{t}^{-1}\right)}^{T}\circ \varphi_{t}\left(x_{i}\right)d\varphi_{s}^{T}\left(x_{i}\right)\nabla \left(\text{div}\;u_{s}\right)\left(x_{i,s}\right)ds. \end{array}$$

Differentiating requires the identity $(d/dt)\left({\left(d\varphi_{t}^{-1}\right)}^{T}\circ \varphi_{t}\right)=-{\left(du_{t}\right)}^{T}\circ \varphi_{t}{\left(d\varphi_{t}^{-1}\right)}^{T}\circ \varphi_{t}$ (see (B.5) below):

(B.4)
$${\dot{p}}_{i,t}^{x}=-{\left(du_{t}\right)}^{T}\left(x_{i,t}\right)({\left(d\varphi_{t}^{-1}\right)}^{T}\left(x_{i,t}\right)p_{i,0}^{x}\;\;-p_{i,0}^{w}w_{i,0}\int_{0}^{t}{\left(d\varphi_{t}^{-1}\right)}^{T}\left(x_{i,t}\right)d\varphi_{s}^{T}\left(x_{i}\right)\nabla \left(\text{div}\;u_{s}\right)\left(x_{i,s}\right)ds)\;\;-p_{i,0}^{w}w_{i,0}{\left(d\varphi_{t}^{-1}\right)}^{T}\left(x_{i,t}\right)d\varphi_{t}^{T}\left(x_{i}\right)\nabla \left(\text{div}\;u_{t}\right)\left(x_{i,t}\right)\;\;=-{\left(du_{t}\right)}^{T}\left(x_{i,t}\right)p_{i,t}^{x}-p_{i,t}^{w}w_{i,t}\nabla \left(\text{div}\;u_{t}\right)\left(x_{i,t}\right),$$

where the last equality uses (B.2), $w_{i,0}p_{i,0}^{w}=w_{i,t}p_{i,t}^{w}$. The remaining identity follows $(d/dt)\left({\left(d\varphi_{t}\right)}^{-1}d\varphi_{t}\right)=0$ implying

(B.5)
$$\begin{array}{l} \frac{d}{dt}{\left(d\varphi_{t}\right)}^{-1}=-{\left(d\varphi_{t}\right)}^{-1}\left(d{\dot{\varphi }}_{t}\right){\left(d\varphi_{t}\right)}^{-1}=-{\left(d\varphi_{t}\right)}^{-1}du_{t}\circ \varphi_{t}\\ \;=-\left(d\varphi_{t}^{-1}\right)\circ \varphi_{t}du_{t}\circ \varphi_{t}. \end{array}$$

The optimal control (18) is written in the endpoint $\left(p_{1}^{x},p_{1}^{w}\right)$ with the boundary conditions (20b). A similar argument as above gives the t=1 boundary corresponding to the first part of (18b) for t=1. For $p_{i,t}^{w}$ then constancy $p_{i,t}^{w}w_{i,t}=p_{i,1}^{w}w_{i,1}$ with $\left|d\varphi_{t,1}\right|\left(x_{i,t}\right)=\left|d\left(\varphi_{1}\circ \varphi_{t}^{-1}\right)\right|\left(x_{i,t}\right)=\left|d\varphi_{1}\right|\left(x_{i}\right)\left|d\varphi_{t}^{-1}\right|\left(x_{i,t}\right)$ gives the second half of (18b) for t=1:

(B.6)
$$p_{i,t}^{w}=\frac{p_{i,1}^{w}w_{i,1}}{w_{i,t}}=\frac{p_{i,1}^{w}\left|d\varphi_{1}\left(x_{i}\right)\right|}{\left|d\varphi_{t}\left(x_{i}\right)\right|}=p_{i,1}^{w}\left|d\varphi_{t,1}\right|\left(x_{i,t}\right).$$
